# The Adverse Effects of Electronic Cigarettes on Oral Health: Where Do We Stand?

**DOI:** 10.3390/dj14100652

**Published:** 2026-10-08

**Authors:** Mahmoud Rouabhia

**Affiliations:** Groupe de Recherche en Écologie Buccale, Faculté de Médecine Dentaire, Université Laval, 2420 Rue de la Terrasse, Québec, QC G1V 0A6, Canada; mahmoud.rouabhia@fmd.ulaval.ca

**Keywords:** electronic cigarettes, aerosol, vaping, oral health, oral microbiota, gingival epithelial cells, oral mucosa, inflammation, reactive oxygen species (ROS), DNA damage

## Abstract

**Background/Objectives**. Electronic cigarettes allow smokers to consume nicotine, a highly addictive substance, without burning tobacco. Since their commercialization in early 2004, electronic cigarettes have become popular in the Asian market and then worldwide. Initially conceived as an alternative to combustible smoking, they have gradually established themselves as a mass consumption product, raising both hopes for the reduction of the risks caused by burned cigarettes and concerns about their use among non-smokers, especially young people. Currently, because of their availability, accessibility to non-smokers, including young people, and their manageability, electronic cigarettes are causing more concern than hope among health professionals. This narrative review aimed to synthesize current evidence on the different chemical components generated by electronic cigarette devices and their effects on oral health. We specifically sought to (i) compile and discuss the available evidence regarding the effects of each chemical component in the e-cigarette aerosols on oral health outcomes, and (ii) feature the risks associated with e-cigarette use and how to ovoid/prevent them. **Methods**: We reviewed relevant English-language literature on electronic cigarette design, use, chemical generation, and adverse effects on oral health through targeted searches of PubMed, Elsevier, Oxford Academic, BioMed Central, MDPI, Scopus, and Google Scholar from 2008 to 2026. Evidence was narratively synthesized to shed light on different oral health concerns reported following electronic cigarette use. **Results:** This narrative review highlights several adverse effects of electronic cigarettes on oral health, including functional deregulation of the soft (tongue, gums, salivary glands) tissues, hard tissues (teeth, dental restorative materials), and the oral microbiota. These effects were attributed to the different chemicals generated when electronic cigarettes heat-vape e-liquids. These chemicals damage oral tissues/cells, leading to apoptosis, oxidative stress, and DNA damage that may lead to oral cancer. These effects may reduce oral cell growth and increase tissue inflammation, which may delay wound healing. The reviewed studies identified several limitations, including the need for additional investigations involving electronic cigarette use and long-term follow-up. **Conclusions:** The available in vitro and in vivo studies clearly show the potential harm of vaping on oral health. This review offers a good overview to electronic cigarette users, oral health professionals, and scientists about the potential oral harm effects of electronic cigarettes. The available studies suggest the need for oral health professionals’ support in providing information and awareness about the harmful effects of electronic cigarettes. Thus, the health system and professional orders should develop information and awareness tools to help oral health professionals in their interventions with e-cigarette users.

## 1. Introduction

Electronic cigarettes went through several generations (Table 1). The first model, called the first generation (cigalike), was inspired by a combustible cigarette in terms of shape and use. This cigalike, designed in 2002, was intended to be an effective model for smokers who wish to reduce their consumption of combustible cigarettes while maintaining the tobacco gesture and access to nicotine on which they are dependent [1]. It was developed using a piezoelectric element and a non-combustion heating resistor system to vaporize a nicotine-containing liquid at low concentrations [1,2]. This system became available to Chinese consumers in 2004 and later (2006–2007) in Europe, North America, and other regions of the world [1]. This first generation is most often for single-use, with a low-power, non-rechargeable battery [1,3].

The first generation of electronic cigarettes very quickly underwent technological improvements, leading to the second generation (vape pens). This generation features an efficient heating system, enabled by a more powerful battery that allows the production of large quantities of aerosol, according to the user’s wishes. The second-generation device has a large tank for e-liquid storage, providing space and the ability to customize e-liquid constituents [1,4].

The evolution of electronic cigarettes continued, leading to the design of third- and fourth-generation devices. The third generation, known as mods, refers to larger electronic cigarette models than those of the first and second generations. It allows users to adjust the power and voltage to their preferences. With this third generation, electronic cigarettes increase the nicotine content of the aerosol due to their high-temperature heating system (up to 230 °C), enabled by more powerful batteries. The fourth generation, or “pod,” is a compact model, carrying fast electronic chipsets. This model offers an intense vaping experience [5]. The electronic cigarettes of this fourth generation are refillable and equipped with replaceable liquid tanks, offering a practical, discreet, and user-friendly model, especially for young people [6].

## 2. Materials and Methods

We conducted electronic search from January to August 2026. Only original studies published in English were considered. We searched for relevant scientific articles indexed from 2008 to 2026 using the following databases: PubMed, Elsevier, Oxford Academic, BioMed Central, MDPI, Scopus, and Google Scholar. We used a consistent search strategy across all databases. Our search included in vitro, in vivo research, and clinical studies evaluating the impact of e-cigarettes on oral health. We used the following Keywords, in combinations of two or more, to conduct our search. “Electronic cigarette/E-cigarette/Vaping”, “Aerosol”, “E-cigarette Temperature”, “Power of e-cigarettes”, “Coil Temperature”, “e-liquid”, “Thermal decomposition”, “Electronic Cigarette Flavors”, “Formaldehyde”, Acetaldehyde”, “Acrolein”, “Hydroxy acetone”, “Acetol”, “Hydroxy propanone”, “Methyl-ethanedial”, “Hemipetals/Hemiacetal formaldehyde”, “Nicotine N-oxide/NNK,/4-(Methylnitrosamino)-1-(3-pyridyl)-1-butanone”, “DNA damage”, “N-nitrosonornicotine/NNN”, “Diacetyl”, “Saliva/Salivary glands” “Oral toxicity” “Inflammation/Cytokine”, “Oral microbiota/Oral dysbiosis/biofilms”, “Oral Health”, “Teeth/Tooth decay”, “Restoration Materials/Dental Restoration”, “Language/Lingual mucosa”, “Taste buds”, “Gum tissue/Gum cells”, “Oral Cancer”, “Periodontitis”. We screened systematic reviews and meta-analyses to identify original studies not found in the initial search. Selected articles were screened by reading the abstract to confirm their link to vaping and its consequences for oral health. The selected articles were read in full to collect pertinent information for inclusion in this review. These articles include the period of 2013 to 2027.

## 3. Literature Analysis

### 3.1. Why Was the Electronic Cigarette Developed?

Because of the harmful health effects of tobacco use, smokers, health care professionals, and regulatory agencies were looking for alternatives to tobacco to eliminate as many toxic products as possible. Previous tobacco smoke replacement initiatives included nicotine substitutes, pharmacotherapies, support for smoking cessation, and related interventions [7,8,9]. However, despite the effectiveness of these strategies, the cessation of cigarette use among smokers remains too low [10,11,12]. In this context, the search for less harmful alternatives to combustible cigarettes has become a public health priority. In this context, researchers designed the electronic cigarette model to reduce the harms of combustible tobacco [13].

The first intended goal in developing electronic cigarettes was to reduce the harmful effects of the chemicals present in cigarette smoke [14].

The second intended goal was to help with smoking cessation [15]. These objectives were based on effective accessibility to nicotine through a model comparable to standard cigarettes. Historically, the electronic cigarette was originally developed as a less harmful alternative to smoked tobacco, intended primarily for adult smokers addicted to nicotine. However, current studies show that electronic cigarettes are used more by young smokers and non-smokers [16]. Other studies report that electronic cigarettes are not as safe as we believe [17,18].

### 3.2. The Consequences of Electronic Cigarettes on Oral Health (Figure 1)

Due to technological advances, electronic cigarettes are less likely to cause physical incidents, such as overheating or explosions, during use or storage. However, the products used to generate aerosols, and the constituents of these aerosols can significantly affect oral health. Indeed, during vaping, the first recipient site of the aerosol generated by an electronic cigarette is the mouth. Thus, aerosol from an electronic cigarette comes into direct contact with all components of the oral cavity, i.e., soft tissues (gums, tongue), teeth, restorative materials, the oral microbiota, and saliva/salivary glands.

**Figure 1:**
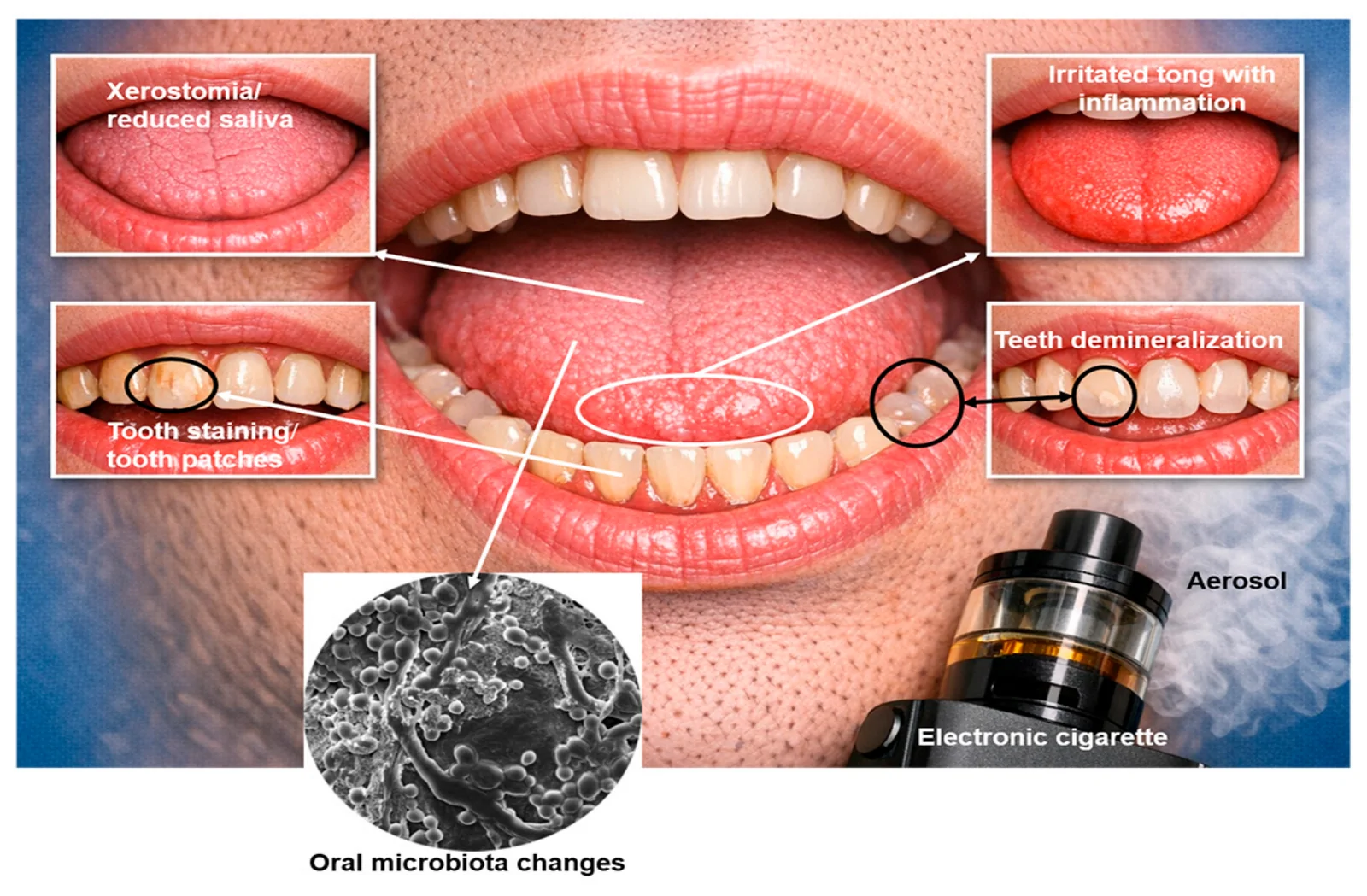
Summary of the potential effects of electronic cigarettes on oral health. Summary of the potential effects of electronic cigarettes on oral health. Electronic cigarette aerosols tend to create a more inflammatory, acidic, and microbiologically unbalanced oral environment. This environment could promote gum disease, periodontal disease, and possibly oral mucosal lesions. Certain photos in this figure were generated by artificial intelligence (Microsoft 365 Copilot, Copilot Chat (secure/protected version) via the Microsoft 365 license,).

### 3.3. The Effects of the Electronic Cigarette on Saliva/Salivary Glands

Several studies have shown that electronic cigarette use impairs salivary gland function, manifested by reduced parasympathetic stimulation and decreased secretory activity of the parotid, submandibular, and sublingual glands [19,20]. Most often, electronic cigarette users suffer from a decrease in resting salivary flow and an increased prevalence of subjective xerostomia (dry mouth) [21,22]. Also, most electronic cigarette users have a lower basal salivary flow rate compared to non-smokers [23]. Even when comparing saliva production between conventional and electronic cigarette smokers, the reduction in saliva production is greater with electronic cigarettes [24,25]. Therefore, one of the first warning signs of electronic cigarettes’ dangers to oral health is reduced saliva production. Another important consequence is altered saliva composition among electronic cigarette users. Biochemical analyses showed a significant increase in lipid peroxidation products (malondialdehyde, 4-hydroxynonenal) due to oxidative stress and altered salivary antioxidant systems caused by dysregulation of salivary gland cells [26,27]. Qualitative analyses of the saliva of electronic cigarette users reveal more acidic saliva (pH up to 5.5) [19,23,28] and more viscous saliva, reducing the lubrication and self-cleaning functions of saliva [29]. Electronic cigarettes also disrupt the innate immune component by diminishing saliva’s antimicrobial and antioxidant capacity [26,28]. These changes in saliva and the salivary glands caused by electronic cigarettes could increase the risk of oral diseases [30].

### 3.4. The Effects of Electronic Cigarettes on the Oral Microbiota

While vaping, the aerosol fills the oral cavity and encounters the oral microbiota. This microbiota is a microbial consortium composed of bacteria, fungi, viruses, and archaea living in dynamic equilibrium with the host. This contact with the aerosol can disrupt this balance. Available studies show that electronic cigarette use decreases microbial diversity and leads to an imbalance between commensal and pathogenic bacteria [31,32]. Studies have shown that vapers exhibit reduced levels of certain bacteria associated with oral health (e.g., Streptococcus sanguinis) but increased levels of periodontopathogenic bacteria, such as *Porphyromonas gingivalis*, *Fusobacterium nucleatum*, and *Prevotella* spp. [32,33], causing dysbiosis of the oral microbiota. Such dysbiosis can increase the risk of cavities, periodontal disease, opportunistic infections, such as oral candidiasis, and chronic inflammation of the oral mucosa. These consequences are attributable to greater microbial virulence in vapers. Indeed, several studies reported that electronic cigarette aerosols increase the expression of virulence genes in pathogenic bacteria in the mouth [34,35]. They also increase bacterial adhesion to various surfaces in the mouth (tooth, soft tissue, and restorative materials) [31,32]. This strong adhesion capacity promotes dental plaque (biofilm) formation, protecting bacteria from the immune defense system and antimicrobial agents [36].

### 3.5. The Effects of the Electronic Cigarette on the Teeth

Teeth are another important part of the mouth that can be exposed to electronic cigarette aerosol. When vaping, all teeth are likely to be covered with the aerosol of the electronic cigarette. This aerosol can directly alter teeth, including demineralization and enamel changes. Through the hygroscopic effects of its constituents, aerosols promote dehydration of the tooth surface and reduce natural remineralization mechanisms [37,38]. In addition, at acidic pH levels, reduced salivary flow and more viscous saliva increase enamel demineralization in vapors, which can lead to tooth erosion [39]. Other studies have shown changes in tooth staining [40,41] due to the oxidation of certain aromatic compounds, modifications to the acquired film, and increased bacterial plaque accumulation [40]. These color changes may prompt users to seek more dental care, including dental polishing.

### 3.6. The Effects of the Electronic Cigarette on Dental Restorative Materials

The various materials used for dental restorations (composite resins, glass ionomer cements, ceramics, and resins) are directly exposed to electronic cigarette aerosols. This exposure could compromise the functions of these materials. Current data indicate that aerosols can alter the optical, mechanical, and aesthetic properties of dental restorative materials [42]. Indeed, dental restorative materials exposed to electronic cigarette aerosols showed color changes, particularly in composite resins [38,43,44]. Microhybrid and nanohybrid composites have better color stability compared to macro-filled composites. However, glass ionomer cements are more susceptible to vaping-related color alterations [45,46]. Electronic cigarette aerosols also increase surface roughness of restorative materials, attributed to resin matrix degradation, loss or partial detachment of inorganic fillers, and chemicals generated during vaping [47,48]. These surface modifications of electronic cigarette aerosol-restored materials can reduce surface microhardness, gradually weakening the dental restorative material [49,50]. These effects depend on the material’s composition, the type of e-liquid (with or without nicotine), and the duration of exposure [44,50]. Scanning electron microscopy corroborated these surface modifications. These analyses revealed microcracks and areas of erosion caused by electronic cigarette aerosol [47,51]. These ultrastructural modifications could compromise the surface integrity of dental materials, accelerating their aging.

### 3.7. The Effects of Electronic Cigarettes on the Tongue

The tongue is an essential component of the oral cavity, playing an important role in chewing, swallowing, phonation, and taste. It is covered by innervated, highly vascularized mucosa. When vaping, the tongue is directly exposed to the aerosols of the electronic cigarette, which could cause several changes to it. Clinical and observational studies reported an association between vaping and symptoms of lingual irritation, including burning, itching, discomfort, and pain [52,53]. These effects stem from solvents such as propylene glycol (PG), which locally dehydrates the mucosa, and nicotine, a vasoconstrictor that can alter lingual microcirculation [23]. These effects promote chronic mucosal inflammation, which is vulnerable to mechanical trauma and lingual infections [54]. Vaping can also alter taste perception, including decreased sensitivity to sweet, salty, or bitter flavors, as well as dysgeusia (abnormal metallic or other tastes) [55]. It should be noted that prolonged use of electronic cigarettes can lead to persistent taste alterations [56]. Vaping is also frequently associated with the appearance of a suboral tongue (whitish or yellowish lingual coating), resulting from the accumulation of keratinous, bacterial, and food debris [32,33], which promotes halitosis, which is very common in electronic cigarette users [57]. Finally, a hairy black tongue has been reported among electronic cigarette users [58,59]. This manifestation is associated with certain flavors and thermal degradation products of e-liquids that adhere to the lingual surface and become trapped in hypertrophied thread-like papillae, causing the tongue to appear dark [59].

### 3.8. Effect of the Electronic Cigarette on the Periodontium

The periodontium comprises specialized tissues, including the gum, periodontal ligament, root cementum, and alveolar bone. These structures support and maintain the teeth in the jawbones. As the oral cavity is the primary site of exposure to e-cigarette aerosols, periodontal tissues are directly affected by the chemicals generated during vaping [60,61]. Human oral mucosa, part of the periodontium, plays an important role as a mechanical and immunological barrier against microbial and chemical aggressions. It is also one of the oral tissues most exposed to the aerosol from electronic cigarettes. This exposure could compromise important functions of gingival epithelial tissue [62,63] (Figure 2). Several studies have shown increased levels of inflammatory mediators, including interleukin-1β (IL-1β) and β-transforming growth factor (TGF-β), in the saliva of electronic cigarette users [63,64,65]. Electronic cigarette use is associated with a decrease in gingival blood perfusion at the oral mucosa, which can cause a decrease in clinical gingival bleeding caused by impaired microcirculation, resulting in a false impression of periodontal health [66]. These microcirculatory alterations can slow tissue repair mechanisms and promote the silent progression of periodontal disease [67]. Indeed, several studies reported alterations in the adhesion and proliferation of gingival epithelial cells [68,69], as well as in gingival fibroblasts, and in their ability to produce the extracellular matrix that forms connective tissue [70,71]. These adverse effects could have important consequences on healing after periodontal or surgical care, postoperative complications, and reduced tissue stability around teeth and implants [72]. Vaping is also associated with an increased risk of gingival recession and clinical attachment loss [73]. Other oral mucosa tissues, including cheek and palate may suffer electronic cigarette harm effects as they are also first in contact with the aerosols. These studies suggest that electronic cigarette aerosols may cause persistent subclinical inflammation and decreased vascularization, which would compromise oral tissue healing, promoting periodontal disesases.

### 3.9. The Effects of Electronic Cigarette Aerosols on Human Cheek or Palate

In addition to its effects on the gums, e-cigarette aerosol can affect other mucous membranes, including the jugal mucosa (cheek) and palate. In early 2018, a study by Bardellini et al. (2024) reported an increased prevalence of lesions in the oral mucous membranes in vapers, suggesting effects of e-cigarette aerosol on oral mucous membranes, including the cheek and palate [74]. These observations are supported by in vitro studies using a gingival tissue model, which show that repeated exposure to electronic-cigarette aerosol disrupts epithelial structure and decreases the production of basal membrane proteins, including laminin and Type IV collagen [64]. Clinical studies confirm these in vitro observations. Indeed, Carvalho et al., (2024) described the appearance of a white plaque located at the level of the posterior hard palate in a vaper. Histopathological examination revealed hyperparakeratosis without dysplasia, suggesting an adaptive epithelial response to repeated exposure to e-cigarette aerosols [28]. In a clinical case, Santo et al., (2024) report that an e-cigarette user for one year presented to the oral medicine department. She wishes stomatitis with pain, eating difficulties and drinking consumption. On intraoral examination, white ulcers with yellowish irregular margins were found in several parts of the oral mucosa. Clinical management, including cessation of vaping, led to an improvement in the patient’s oral condition one week after therapy [75].

Also, Kotha et al. (2024), in a case series study, reported that regular e-cigarette use caused ulcers on the oral mucosa. These clinical manifestations were resolved after the cessation of e-cigarette use, suggesting a link between vaping and the onset of episodes of aphthous ulcers [76]. A pilot study evaluating the effects of standard cigarettes and e-cigarettes on the blood flow of the palatal membrane shows minimal impact on palatal blood flow [77]. Reeve et al., (2023) performed histological and transcriptomic analyses on biopsies taken from the oral mucosa of e-cigarette users for about 2 years. They found no histological or transcriptomic abnormalities [78]. However, a recent study reported that e-cigarette users are at risk of developing leukoplakia and mouth ulcers [79]. Overall, these studies suggest that e-cigarette aerosols may harm all oral mucous tissues, including the cheeks and palate. We still need additional clinical longitudinal studies to confirm whether vaping can damage oral mucous membranes.

### 3.10. Electronic Cigarette Use May Promote Oral Cancer

Because electronic cigarette aerosol causes significant ROS production by gingival cells, especially epithelial cells, several in vitro, in vivo, and clinical studies suggest that electronic cigarettes can induce biological mechanisms involved in oral carcinogenesis. Indeed, e-liquids have been shown to modify the behavior of normal oral epithelial and tumor cells by promoting proliferation, cell migration, invasion, and epithelial–mesenchymal transition, a key process in the progression of oral squamous cell cancers [80,81]. These in vitro studies are supported by in vivo studies showing that oral cells obtained by brushing from e-cigarette users’ express genotoxicity markers and DNA adducts [82,83]. These observations are supported by a recent transcriptomic study of oral epithelial cells from vapers showing that electronic cigarette use is associated with substantial transcriptomic perturbations [84] that may lead to oral cancer development. It is important to note that to date, there is no direct clinical evidence confirming the implication of electronic aerosols in oral cancer, as the available studies are mainly on early biomarkers of carcinogenesis collected from in vitro and ex vivo studies [80,81,82,83,84]. Overall, electronic cigarettes could lead to oral cancer development. However, additional clinical studies are needed to confirm this possibility.

### 3.11. Electronic-Cigarette Chemicals That May Harm the Mouth

The electronic cigarette is a device composed of an energy source (a battery), a tank to store e-liquid, and an atomizer that generates aerosol. To generate this aerosol, the battery heats the e-liquid to a high temperature (Table 2). This liquid is most often made up of the following:

Humectant (PG and glycerin). These chemicals are used in different proportions (50/50%, 80/20%, 30/70%, etc.) depending on the amount of aerosol the user wants to produce with their electronic cigarette.

Flavors are also found to improve the acceptability of the electronic cigarette by users by reducing the pungent and irritating taste of nicotine when vaping, especially among new users [92]. These flavors are not necessary to operate the electronic cigarette device, but they constitute a behavioral, sensory, and, above all, commercial tool for electronic cigarettes. Users can choose from several hundred flavors (fruity, sweet, menthol, drinks, desserts, etc.). In 2014, more than 7000 distinct flavors were made available to electronic cigarette users [93]. In 2016–2017, more than 15,000 distinct flavors were available [94]. Whether heated or not, chronic contact between the oral components and these aromas can cause significant oral problems [92]. To reduce the health harm that these flavors cause, several countries have regulated their inclusion in e-liquids. In Canada, as of 2021, only tobacco, mint, and menthol flavors are permitted [95]. In the USA, only tobacco and menthol flavors are allowed [96].

Nicotine is one of the main constituents of electronic cigarette liquids. It is the main molecule responsible for tobacco addiction. In e-liquids, nicotine is dissolved in the humectant (PG and vegetable glycerin (VG)). The e-liquids are offered with varying nicotine contents to satisfy users, especially in the context of smoking cessation. These concentrations range from 0 mg/mL (without nicotine) to 20 mg/mL. Various countries, including Canada and European countries, regulate the maximum concentration [97]. These concentrations are organized into intervals ranging from 0 mg/mL for users who wish to vape without nicotine; 3 to 6 mg/mL for light smokers and those who wish to reduce the use of standard cigarettes; 9 to 12 mg/mL for electronic cigarette users who smoke standard cigarettes moderately; 18 to 20 mg/mL for those who smoke standard cigarettes heavily [98].

However, several analyses have shown differences in nicotine levels between what is advertised and what is present in e-liquids. These studies indicate that several analyzed e-liquids had concentrations (higher or lower) that differed from those reported by the manufacturers [99,100]. Recent chromatographic analyses confirm significant variations across brands, batches, and storage conditions. These differences raise public health and regulatory compliance issues [101,102]. Nicotine can be found in two forms. The traditional, more alkaline form provides a marked “hit” sensation, while nicotine salts have a lower pH due to the presence of acid, most commonly benzoic acid. These nicotine salts appear to offer smoother inhalation and faster absorption [98].

### 3.12. Temperatures Generated by Each Generation of Electronic Cigarettes

In this section, we refer to the coil temperature when generating the aerosol. The electronic cigarette generates an aerosol by heating e-liquid. This heating system is more powerful across the first to fourth generations of electronic cigarettes (Table 2).

The first generation (“cigalike”) uses a weak, non-adjustable battery; several studies show its resistance reaches temperatures between 100 °C and 200 °C under normal use [85]. At times, the temperature can reach 250 °C, especially when the e-liquid supply is insufficient (“dry puff”).

The second-generation device (vape pens) has a more powerful battery, allowing it to reach higher resistance temperatures. These temperatures can range from 150 °C to 300 °C [86,91].

The third-generation device (mods) has a more powerful heating system that can generate resistance temperatures ranging from 190 °C to 350 °C or higher [87].

The fourth generation (pods) includes a sub-ohm system capable of high-power operation. Several studies report that, under well-controlled conditions, pods can generate temperatures ranging from 250 °C to 400 °C [89,90,91]. To control maximum temperatures, these devices use a system comprising nickel, titanium, or stainless steel to limit the temperature to 200 °C or 300 °C [89]. We note that temperature varies widely from one generation of electronic cigarettes to another. These temperatures (100 °C to 350 °C) can cause the formation of potentially toxic substances during vaping.

### 3.13. Effect of Electronic Cigarette Heating on E-Liquid Product Decomposition

#### 3.13.1. Electronic Cigarette Heating Affects PG

PG (C_3_H_8_O_2_) is used as an aerosolization carrier in many industrial, pharmaceutical, and food applications. It is a water-miscible liquid with a boiling point of approximately 188 °C [103,104]. In electronic cigarettes, PG is used as a humectant to deliver nicotine. PG can undergo chemical transformations depending on temperature, heating time, and oxygen exposure (Table 3). At approximately 180 °C, PG partially evaporates without pyrolysis [105], but it can undergo slow oxidation that forms trace amounts of carbonyl compounds, including formaldehyde (FA) and acetaldehyde [106,107]. Beyond the boiling point, thermal decomposition of PG becomes important due to dehydrogenation, partial oxidation, and fragmentation of the carbon backbone [108]. These different decomposition mechanisms yield distinct chemical compounds, including FA, acetaldehyde, and acetone [106]. Heating the metal surfaces (nickel, chromium, stainless steel) of the electronic cigarette device can accelerate PG degradation, even at low temperatures [105,109,110].

#### 3.13.2. Electronic Cigarette Heating Affects VG

In e-liquids, VG is used as a dense aerosol-generating humectant. VG (glycerol, 1,2,3-propanetriol, C_3_H_8_O_3_) is a viscous tri-alcohol miscible with water. It has good thermal stability with a boiling point of about 290 °C [113]. However, VG can undergo significant chemical transformations depending on temperature, oxygen exposure, and the metal in the electronic cigarette device (Table 4). At temperatures between 100 °C and 180 °C, such as those generated by first-generation electronic cigarette devices, VG undergoes partial evaporation and nebulization into microdroplets, without changing its molecular structure [85,113].

At these temperatures, oxidation can also occur, generating FA and acetaldehyde [111,114]; both can harm the oral cavity. At temperatures ranging from 180 °C to 250 °C, as generated by second- to third-generation electronic cigarette devices, the decomposition of VG leads to the formation of, among other things, acrolein (2-propenal) [114]. At temperatures above 250–300 °C, VG undergoes partial pyrolysis, forming acrolein and reactive aldehydes and dicarbonyls [115,116,117,118]. Metal resistances (stainless steel, nickel, chromium) used to control temperature increases can increase aldehyde formation, even at low temperatures [119,120]. Therefore, the use of VG when vaping can be a significant source of potentially toxic chemicals for vapers.

**Table 4:** Chemicals generated by thermal degradation of VG during vaping.

Electronic Cigarette Generation	Temperature of the Resistor	Chemicals from VG	Scientific References
1st generation: cigalikes, cartomizers	<5 W ≈100–200 °C (humid conditions)	Glycerol intact in the majority; traces of formaldehyde, acetaldehyde (via hemiacetals).	[85,112]
2nd generation: eGo, clearomiseurs	5–10 W ≈150–300 °C (wet to partially dry)	Acrolein, formaldehyde, acetaldehyde, acetone, formic acid and acrylic	[85]
3rd generation: Variable Power Mods	20–100 W ≈200–350 °C	Acrolein (Increased with VG), formaldehyde, glycidol, acetaldehyde	[87,108]
4th generation: Temperature Control (TC) Mods	6–15 W ≈180–250 °C)	Acrolein, formaldehyde (up to cigarette-like levels), acetaldehyde	[89]
Extreme use/dry hit: Dry wick, strong power	>350–1000 °C (locally)	Acrolein (high), formaldehyde, acetaldehyde, CO, multiple carbonyl compounds	[87,115]

#### 3.13.3. Electronic Cigarette Heating Affects Nicotine

Nicotine is one of the compounds delivered by electronic cigarettes to satisfy the desire of smokers. In such conditions, nicotine can undergo chemical transformations due to electronic cigarette heat. Generally, nicotine’s boiling point is approximately 247 °C [121]. However, nicotine’s thermal stability depends on temperature, oxygen presence, and the medium in which it is dissolved (tobacco, e-liquid, nicotine salts) [122]. In electronic cigarettes, the first factor that can contribute to nicotine transformation is temperature (Table 5). Several studies show that at temperatures below 200 °C, nicotine can be vaporized into aerosols without chemical transformation [121]. However, even at this low temperature, if nicotine contacts oxygen, small amounts of by-products, including nicotine N-oxide or nicotyrin, form [123,124]. When nicotine is heated between 200 °C and 350 °C, several chemical by-products form, including myosmin, nicotyrin, pyridines, and picolines [125]. Above 350 °C, nicotine pyrolyzes, breaking down [126,127] by rupture of the C–N and C–C bonds and forming several nitrogenous and aromatic molecules, including cyanide, hydrogen, and pyridinyl radicals. These different molecules can cause significant health problems [126].

#### 3.13.4. Consequences of Flavor Heating by Electronic Cigarettes

Flavors are added to e-liquid to improve its taste, thereby promoting the use of electronic cigarettes. Flavors are aromatic aldehydes (vanillin, benzaldehyde, cinnamaldehyde), ketones, esters, lactones, and terpenes (limonene, linalool) [133,134]. However, the flavor regulations do not cover the use of flavors after heating and inhalation. Adding flavors to e-liquids exposes them to different temperatures that can cause different transformations (Table 6). In first-generation e-cigarettes, which reach temperatures ranging from 100 °C to 200 °C, some flavors transfer directly into the inhaled aerosol, while aromatic aldehydes can undergo chemical reactions [133,135]. Aldehyde flavors (vanillin, benzaldehyde, cinnamaldehyde) react with PG and VG, forming hemiacetals and acetals in the liquid phase and in the aerosol during vaporization [136,137]. At temperatures between 200 °C and 250 °C, as generated by the second generation of e-cigarettes, most flavors lead to the formation of toxic aldehydes in the aerosol [138,139]. Flavors may be the main source of toxic products such as FA, acetaldehyde, and acrolein, surpassing those from PG and glycerin [138]. At higher temperatures (≥250–300 °C), such as those emitted by the third and fourth generations of e-cigarettes, flavors, especially sugar, creamy, or pastry flavors, undergo partial pyrolysis, resulting in the formation of FA, acrolein, acetoin, and diacetyl, 2,3-pentanedione [140,141]. Inhalation of these chemicals causes significant changes in oral cells, which can lead to serious health problems [68,142,143,144,145].

## 4. Diversity of Chemicals Formed During Vaping

Under the heat generated by electronic cigarettes, e-liquids form several potentially dangerous chemical compounds (Table 7) that can harm oral and general health. This heating causes pyrolysis, oxidation, dehydration, and catalysis of the e-liquid’s constituents, leading to the formation of a wide range of common and unique secondary compounds in the inhaled aerosol [85,139,149]. The table below summarizes the chemical compounds formed after vaping a liquid containing nicotine and a flavor.

## 5. How Do These Aerosol-Derived Chemical Compounds Alter Oral Health (Figure 2)?

### 5.1. Effects of FA Generated by E-Cigarettes on Human Oral Cells

FA is a highly reactive aldehyde. Human cells produce it in small amounts as a by-product of the “one-carbon cycle,” which involves vitamin B9 and facilitates the manufacture of DNA and amino acids [163,164]. FA is therefore a biochemical regulator of gene expression at the cellular level; enzymes, including ALDH2 and ADH5, remove excess FA. The latter rapidly converts FA into formate, a harmless substance [163,164]. However, FA is highly reactive with various cellular constituents (DNA, proteins, and lipids), leading to significant cytotoxicity [165]. Through this toxicity, FA deregulates DNA metabolism, proliferation, and repair systems [163,166,167]. These alterations increase cells’ sensitivity to damage, which can promote cancer development [166].

During vaping, FA concentrations can vary with use conditions (temperature, potency, inhalation behavior) and may reach biologically active levels capable of causing DNA damage in oral cells [168,169]. Gum epithelial cells are the first to encounter the FA formed during vaping. Gingival cells exposed in vitro to e-cigarette aerosol show double-stranded DNA breaks, high micronuclei levels, and accumulated genotoxic damage [82,169]. A few studies report that gingival cells from e-cigarette users exhibit significantly higher levels of DNA damage than oral cells from non-e-cigarette users [168,169]. The FA generated by vaping causes oxidative stress in oral cells [70,170,171]. Indeed, several in vitro studies show increased oxidative stress, significant membrane lipid peroxidation, and decreased cellular antioxidant defenses after exposure to electronic cigarette aerosol, likely due to the presence of FA [169,170,171,172]. Oxidative stress activates inflammatory signaling pathways, leading to chronic overproduction of pro-inflammatory cytokines in gingival and oral tissues [64,173]. Oxidative stress induced by paraformaldehyde can cause cell death via apoptosis/necrosis. Several in vitro studies report a significant decrease in gingival epithelial cell viability after exposure to e-cigarette aerosols [68], as well as tissue disorganization of the gingival mucosa [64]. These cytotoxic activities result in cell death by apoptosis/necrosis, respiratory dysfunction, and altered cellular metabolism [68,174]. Repeated exposure of oral tissues to e-cigarette/FA aerosol can cause mutations and delay DNA repair mechanisms, leading to the development of oral cancer [175,176]. Therefore, FA exposure from e-cigarettes can disrupt the balance and health of oral tissues.

### 5.2. Effects of Acetaldehyde Generated by E-Cigarettes on Human Oral Cells

Acetaldehyde is a toxic compound that interacts with various cellular macromolecules [177,178], leading to DNA damage and genomic instability that may promote cancer development [178,179]. E-cigarette aerosol contains acetaldehyde, a biologically active reactive aldehyde [180]. After vaping, this acetaldehyde can dissolve in saliva and come into direct contact with the oral epithelium, resulting in chronic local exposure that may affect oral cells [152]. A mixture of aldehydes, including acetaldehyde, in e-cigarette aerosol has been reported to cause significant damage to oral epithelial cells, leading to increased micronuclei, DNA breaks, and an accumulation of genetic alterations [169]. Examination of oral cells taken from e-cigarette users revealed a proportion of cells with DNA damage 2.6 times higher than that observed in non-vapers [169,177]. This damage may result from reactive oxygen species (ROS) generated when cells/tissues are exposed to e-cigarette aerosol. These ROS cause oxidative stress that affects several components, including oral cell mitochondria and the cytoskeleton [169]. E-cigarette aerosols decrease cell viability by damaging cells [181]. They promote chronic inflammation of oral tissues, which can lead to oral pathologies, including periodontitis [67,182]. With these disturbances in cells and oral tissues, chemicals such as acetaldehyde generated by e-cigarettes could promote an oral cell and tissue changes [183,184]. The acetaldehyde generated by e-cigarettes is therefore an important factor in toxicity for human oral cells that can lead to several oral pathologies.

### 5.3. Effects of Acrolein Generated by E-Cigarettes on Human Oral Cells

Acrolein is an aldehyde present in the environment, produced by cigarette smoke and exhaust gases [185,186]. Acrolein is highly electrophilic. This property enables it to interact rapidly with cellular constituents, leading to several dysregulations [187]. The aerosol of the e-cigarette has been shown to contain acrolein [180]. When it encounters oral cells, the acrolein-rich aerosol can induce multiple changes in these cells and their physiological functions. Indeed, because it is soluble in an aqueous medium, acrolein can reach all components of the mouth (gums, teeth) during vaping [188,189]. Repeated use of e-cigarettes would lead to chronic exposure to oral acrolein components that can cause several oral pathologies. Several studies report that acrolein causes DNA damage, leading to genomic instability. This instability could explain the sharp increase in ROS and NADPH oxidase, and the imbalance in cellular redox, in oral cells exposed to e-cigarette aerosol [190,191]. These disturbances disrupt oral cell function, including proliferative capacity. Indeed, acrolein has been reported to inhibit the adhesion and multiplication of human gingival fibroblasts. Morphological changes and cell death accompany this reduction in cell growth, driven by apoptosis/necrosis [180,192]. Through acrolein, e-cigarettes promote chronic inflammation [114] and can potentially cause oral cancer [82,175]. Acrolein activates several oncogenic signaling pathways, including the epidermal growth factor receptor (EGFR) pathway, which is involved in oral squamous cell carcinoma development [193]. The association between EGFR activation, oxidative stress, DNA damage, and chronic inflammation may promote oral cancer development [175,193].

### 5.4. Effects of Hydroxyacetone Generated by E-Cigarettes on Human Oral Cells

Hydroxyacetone is a carbonyl compound used in various cosmetic products, including self-tanners, and it has recently been identified as a degradation product in e-cigarette aerosols [189]. High-temperature heating of e-liquids in e-cigarettes generates high concentrations (up to ~12 mg/mL) of hydroxyacetone in aerosols [194]. When vaping, there is repeated exposure of oral cells to aerosol/hydroxy acetone, which could cause multiple disruptions of their cellular/tissue functions. Exposure to hydroxyacetone has been reported to decrease oral cell viability and proliferation, potentially delaying oral tissue regeneration [195]. Hydroxyacetone increases ROS and hydrogen peroxide concentrations and disrupts oral cell energy metabolism [90,196]. Hydroxyacetone also affects cell structure by destabilizing cytoskeletal structures, including cytoplasmic and perinuclear filaments, causing a loss of cell shape and decreased adhesion of oral cells [197]. These effects may reduce the barrier function of the oral epithelium, thereby promoting infections that can cause periodontal disease [198]. It is even possible that hydroxyacetone causes DNA damage and increases the frequency of mutations that can cause the development of oral cancer in exposed human cells [199]. In summary, the cytotoxic, genotoxic, and prooxidant effects of hydroxyacetone can significantly harm oral health by weakening innate defenses, slowing wound-healing mechanisms, and increasing the risk of precancerous lesions.

### 5.5. Effects of Methylglyoxal Generated by E-Cigarettes on Human Oral Cells

Methylglyoxal (MG) is a highly reactive compound produced in small quantities during glycolysis, the metabolic activity of the cell. It can also come from external sources, including diet or tobacco consumption [200]. Recently, MG has been identified as a chemical component of e-cigarette aerosols [201]. Because of its high electrophilicity, MG can cause significant damage to human tissues and oral cells [202,203]. While vaping, elevated concentrations of MG have been identified in e-cigarette aerosols. Chemical analyses show that measurable levels of MG are present in aerosols inhaled by users, resulting in direct exposure of oral tissues through dissolution in saliva and prolonged contact [202,204]. It causes, among other things, damage to oral and respiratory epithelial structures by reducing adhesion and cell proliferation, increasing cell death through apoptosis and necrosis, and eliciting an inappropriate pro-inflammatory response [205,206,207]. These effects stem from oxidative stress, as MG generates ROS. These ROS contribute to membrane lipid damage, protein oxidation, and DNA alterations [202,204]. This damage to oral tissues could compromise the mouth’s innate immune defense. DNA damage at the molecular level could even promote the development of oral cancer [194].

### 5.6. Effects of Electronic Cigarette-Generated Hemicetals on Human Oral Cells

Hemicetals are intermediate chemical compounds formed during the reaction between an alcohol and a ketone. In biological systems, they can form spontaneously from reactive carbonyl compounds, especially during the degradation of organic solvents or in environments rich in aldehydes and ketones. Although hemicetals are generally unstable, their transient formation can influence the reactivity and toxicity of carbonyls in human cells [208]. Their role is particularly relevant in chemical exposures, such as aerosols generated by electronic cigarettes, containing carbonyl compounds. Hemicetals form by reacting FA with PG or glycerin [208]. Hemicetals are present in large quantities, sometimes exceeding FA levels in electronic cigarette aerosols. These hemicetals are considered potential vectors of reactive carbonyl compounds toxic to oral cells [112]. After vaping, hemicetals in the aerosol dissolve in saliva, allowing them to come into direct contact with oral tissues [82,114]. Hemicetals affect oral cells through oxidative stress. Several studies report increased ROS levels in cells exposed to e-cigarette aerosols. This oxidative stress reduces oral cell adhesion and proliferation by inducing apoptosis and necrosis [68,198]. Through oxidative stress, hemicetals induce chronic inflammation in the gums, altering innate immune defenses, disrupting the balance of the oral microbiota, and activating inflammatory pathways [114,198]. Hemicetals are considered genotoxic because they cause DNA damage that can lead to oral cancer [82,198]. Therefore, the hemicetals produced by e-cigarettes represent a particular form of indirect chemical toxicity. Although they are less reactive than free aldehydes, their ability to act as reservoirs of toxic carbonyl compounds, including FA, makes them important contributors to aerosol toxicity. This toxicity primarily affects the oral health of electronic cigarette users.

### 5.7. Effects of Nicotine N-Oxide Generated by the Electronic Cigarette on Human Oral Cells

Nicotine N-oxide is a metabolite formed mainly by enzymatic oxidation [209], by heating (vaping), or after inhalation of nicotine [210,211]. Nicotine N-oxide in the aerosol dissolves in saliva and repeatedly contacts oral tissues, potentially causing significant damage to oral health [212,213]. Nicotine N-oxide induces oxidative stress in oral cells by participating in redox cycles that lead to the formation of ROS [214]. These ROS decrease adhesion and growth of oral cells, leading to an imbalance in cellular functions [215]. Nicotine N-oxide increases the oral inflammatory response by promoting the secretion of pro-inflammatory cytokines [216]. These studies suggest that nicotine N-oxide significantly affects the oral epithelial barrier, promoting oral infections and the possible development of oral cancer.

### 5.8. Effects of 4-(Methylnitrosamino)-1-(3-Pyridyl)-1-Butanone (NNK) Generated by E-Cigarettes on Human Oral Cells

4-(methylnitrosamino)-1-(3-pyridyl)-1-butanone (NNK) is a tobacco-specific nitrosamine derived from nicotine and found in cigarette smoke and tobacco products [217,218]. Electronic cigarettes can also generate NNK [219]. The presence of this NNK in e-cigarette aerosol can cause several oral health problems in vapers [219]. Indeed, repeated exposure to e-cigarette aerosols is equivalent to chronic exposure to NNK. This chronic exposure can damage gingival epithelial cells by disrupting the cell cycle and p53 function. This dysregulation can delay or arrest the cell cycle, which can, in turn, delay damage repair and lead to cell death by apoptosis [220]. This damage to oral epithelial cells involves the oxidative stress pathway [221]. NNK is a potent agent that induces oxidative stress in human cells by increasing ROS production. These ROS promote DNA oxidation, lipid peroxidation, and alteration of cellular proteins [222]. This oxidative stress could amplify genotoxic damage by altering oral cell function. NNK can also cause chronic inflammation in the oral tissues by activating several signaling pathways, including NF-κB. This activation would induce the secretion of several pro-inflammatory mediators, causing alterations in gingival tissue [174,175,220]. The maintenance of this inflammatory situation due to repeated exposure to e-cigarette aerosols would promote the development of periodontal disease and oral cancer [174,175,220].

### 5.9. Effects of N-Nitrosonornicotine (NNN) Generated by E-Cigarettes on Human Oral Cells

N-nitrosonornicotine (NNN) is a tobacco-specific nitrosamine, formed primarily by nitrosation of nornicotine, and known to be harmful to health [223,224]. The presence of NNNs in e-cigarette aerosols has been reported [136,225,226]. Through their presence in the aerosol of electronic cigarettes, NNNs can encounter various components of the mouth and cause various damage [136,227]. Indeed, after vaping, NNNs can be deposited in the oral cavity, dissolve in saliva, and come into prolonged contact with the oral epithelium [226]. This repeated exposure can cause oxidative stress to the oral cells. Such stress is thought to cause oxidation of DNA bases, lipid peroxidation of cell membranes, and protein modifications [220]. Among its consequences, NNN disrupts the cell cycle by promoting apoptosis. It can also promote improper cell division, leading to malignant cell transformation [228]. These transformations can cause DNA replication errors, mutations, and genomic instability [220,227], which can lead to cancer development [229]. NNN can also induce an inflammatory response in oral tissues [68,169]. Inflammation is thought to cause overproduction of pro-inflammatory cytokines and deterioration of gingival tissues, leading to oral pathologies such as periodontitis and precancerous lesions [68,230].

### 5.10. Effects of Benzaldehyde Generated by E-Cigarettes on Human Oral Cells

Benzaldehyde (C_6_H_5_CHO) is an aromatic compound widely used in the food, pharmaceutical, and cosmetic industries, especially as a flavoring. Benzaldehyde may also be present in the environment and in some aerosols from electronic devices. Although it is considered relatively safe at low doses, several studies show that benzaldehyde can exhibit cytotoxic effects in human cells at high concentrations or during chronic exposure [231,232]. During vaping, the benzaldehyde generated can be inhaled as an aerosol and come into direct contact with tissues in the oral cavity [148]. After vaping, benzaldehyde deposits in the oral cavity and dissolves in saliva, encountering oral cells [148,233]. Local but repeated exposure to benzaldehyde is likely to damage the oral cavity, particularly in areas directly exposed to the aerosol. Indeed, exposure of human cells to benzaldehyde results in dose-dependent cytotoxicity, including decreased cell viability, altered cell morphology, and growth inhibition [69,234]. These effects are attributed to the aldehyde group, which can induce oxidative stress in oral cells by increasing ROS production [235]. Benzaldehyde affects human cells by oxidizing membrane lipids and altering DNA [236]. Oral cells and tissues repeatedly exposed to e-cigarette aerosol secrete more pro-inflammatory cytokines [64,237]. Chemicals in e-cigarette aerosol, including benzaldehyde, can alter the epithelial barrier, reduce tissue repair capacity, increase local oxidative stress, and promote chronic inflammation. All these factors can compromise oral health.

### 5.11. Effects of Diacetyl Generated by E-Cigarettes on Human Oral Cells

Diacetyl (2,3-butanedione) is a volatile dicarbonyl compound used as a flavoring agent in several food products and in some industrial aerosols. This same product is present in some e-liquids and e-cigarette aerosols [99]. It is most used to achieve butter or cream flavors [162]. Diacetyl is known for its toxic effects on the respiratory system [238]. It interacts with various cellular macromolecules, leading to cytotoxic, prooxidant, and inflammatory effects in oral cells [239,240]. Present in aerosol generated by electronic cigarettes, diacetyl first encounters the various components of the oral cavity, thereby negatively influencing oral health [239]. In contact with cells, diacetyl alters cell morphology and reduces cell viability [241]. It contributes to protein adduct formation, protein structural modification, and loss of enzyme function, which may cause cell stress [242,243,244]. In the long term, these effects could promote the development of epithelial cancer in the mouth and respiratory system [245,246]. Overall, e-cigarette aerosols generate toxic compounds, namely ROS agents, that damage oral cells and tissues.

## 6. ROS Pathway Activation by E-Cigarette Aerosols and Their Consequences on Oral Cells and Tissues

Oxidative stress is one of the main molecular mechanisms responsible for vaping-induced damage to oral tissues. It results from excessive production of Reactive Oxygen Species (ROS) that exceed cells’ antioxidant defense capacity. This ROS increase is triggered by several complementary mechanisms involving constituents in the aerosol generated by the electronic cigarette.

As explained earlier, when the e-cigarette heats the e-liquid, the humectants (PG and VG) and other constituents (nicotine, flavor/flavors) undergo thermal degradation, generating highly reactive compounds, including formaldehyde, acrolein, and acetaldehyde. As shown in Figure 3, these carbonyl molecules directly promote the formation of ROS in oral cells [247,248]. Oral cell contact with vaping-related toxic compounds disrupts the mitochondrial electron transport chain, inducing superoxide anion (O_2_•−) formation. The latter will be converted into hydrogen peroxide (H_2_O_2_) and then into hydroxyl radical (•OH). The accumulation of these reactive species is a major source of intracellular oxidative stress [249,250]. 

One identified mechanism is that compounds in electronic cigarette aerosol increase NADPH oxidase (NOX) activity. These NOXs catalyze the transfer of electrons from NADPH to molecular oxygen in oral cells, among other things, amplifying oxidative stress [144,145]. On the other hand, nicotine, a predominant compound in e-liquids for vaping, interacts with α7 nicotinic receptors (α7-nAChR) expressed by oral epithelial cells, activating several intracellular signaling pathways, including the PI3K/Akt pathway [251,252]; MAPK/ERK [253]; and PKC [254]. Activation of these signaling pathways contributes to NADPH oxidase activation and ROS production [251].

Overproduction of ROS in cells exposed to e-cigarette aerosols inactivates KEAP1, releasing NRF2. NRF2 then binds to the Antioxidant Response Elements (ARE), thereby blocking cellular antioxidant defense [255]. Beyond direct toxicity, ROS activates several signaling pathways involved in oral tissue damage. Indeed, ROS activates the IKK complex, leading to IκB degradation and NF-κB nuclear translocation [256], promoting the secretion of inflammatory mediators such as IL1α, IL6, IL8, and TNFα [257]. These mediators contribute to oral tissue damage and periodontitis [258].

Generated by exposure to e-cigarette aerosol, ROS also contribute to DNA damage by oxidizing nucleic bases, causing single- and double-strand breaks, and promoting DNA adduct formation [259]. It is important to note that e-cigarette aerosols can decrease the efficiency of DNA repair systems, promoting the accumulation of mutations [260] that may lead to oral cancer.

## 7. Limitations of Current Studies Assessing the Oral Health Harms of E-Cigarettes

Despite their abundance and importance, studies evaluating the interactions between electronic cigarettes and their constituents with various components of the mouth have several limitations.

Most current studies are conducted in vitro using human cell lines, and the results are difficult to translate to vapers. Few studies use normal human cells or 3D tissue models that partially replicate vapers’ tissues. Also, exposure time to electronic cigarette aerosols, nicotine doses, humectant mixtures, and flavors are not standardized across studies.

Current studies on electronic cigarettes are not standardized with respect to nicotine concentrations, e-liquid type, flavor, flavor chemical composition, battery power, heating modes, and temperatures. These multiple parameters limit the availability of standard and solid information to be offered to vapers and oral health professionals regarding the harms of e-cigarettes.

Current studies only cover the short- and medium-term effects of e-cigarettes on oral health. To evaluate the effects of e-cigarettes on oral tissues (hard and soft), the oral microbiota, and oral pathologies (cavities, periodontal disease, oral cancer, etc.), researchers need to follow a cohort of e-cigarette users for several decades. These long-term studies will allow us to better understand the effects of e-cigarettes on oral health.

Studies involving human participants also have many limitations. They most often include participants who are previous smokers; participants who combine cigarettes with electronic cigarettes; participants who use e-cigarettes in conjunction with other sources of nicotine, other than standard cigarette smoke. Therefore, these studies cannot identify the specific effects attributable to e-cigarettes. They most often include participants whose hygiene and eating conditions vary across studies. The lack of management of several factors, including sugar and alcohol consumption, is a limitation. Another limitation is the small number of participants; the diversity of use (frequency of use, volume of e-liquid vaped, amount of nicotine used, etc.) limits their scope. Most available studies report results obtained through questionnaires with self-reported responses. As a result, participants may have underestimated or overestimated their actual use, misjudged harms, and so on.

Overall, current studies are highly relevant and suggest possible harms of e-cigarettes to oral health. However, more robust studies that address the limitations listed above are needed. These future studies should focus on comparing the effects of e-cigarettes in vapers versus non-vapers.

Therefore, future studies should include the following:

1. Groups of individuals followed over a long period of time, including people who use e-cigarettes exclusively, tobacco-only smokers, users of both standard cigarettes and e-cigarettes, as well as non-smokers and non-users of e-cigarettes. It is important to consider the age at which electronic cigarette use began.
2. Harmonization of the use of devices, well-defined e-liquids, and well-regulated consumption in time and quantity.
3. It is also important to have a process in place to properly identify and control confounding factors.
4. Participants’ oral health should be monitored over the long term (beyond 15 years).

These suggestions would help better determine the real risks associated with electronic cigarette use. They would also allow better characterization of its effects on oral health and, as a result, more effective prevention and control strategies.

## 8. Conclusions

Based on available in vitro, in vivo, and clinical studies, we conclude that electronic cigarette aerosols interact with different constituents (soft tissues, hard tissues, saliva, oral microorganisms, etc.), leading to dysregulation that may cause oral diseases (Figure 4). Thus, e-cigarettes are not harmless to oral health. Therefore, the electronic cigarettes users must be informed about the potential harms of vaping; use lower doses of nicotine; reduce the use of electronic cigarettes as much as possible; avoid the use of cigarettes and e-cigarettes at the same time; document any changes in oral health (dryness, bleeding, ulceration, cough, etc.) to report them to their dental health professional; maintain adequate oral hygiene while vaping, etc.

The efforts of e-cigarette users to reduce/prevent vaping adverse effects could be supported by assistance from an oral health professional (dentists and oral hygienists). To this end, it would be relevant that oral health professionals systematically ask the patient about their use of electronic cigarettes (frequency, duration, product type, nicotine content) and document this information in their oral health record, inform patients, whether they use electronic cigarettes, that this method of nicotine consumption is not without risk to their oral health, explain to users the possible oral changes (dry mouth, irritation of the mucous membranes, gum inflammation, and increased risk of periodontal disease), monitor the patient more closely for dry mouth, mucosal irritation, gingival inflammation, halitosis, etc., during oral examinations, encourage the patient to have oral exams more frequently, and promote adequate oral hygiene (brushing, interdental cleaning, etc.) among electronic cigarette users.

Ideally, the oral health professional should be involved at multiple levels, including raising awareness of the harms of e-cigarettes, detecting clinical changes, and advising the patient on the consequences of e-cigarette use on their oral health. They should also encourage the vaping patient to adhere to regular follow-up with the oral health professional to reduce the consequences of vaping on oral health, while recommending that they gradually or completely quit smoking and e-cigarette use. For their part, oral health professionals will need support in providing information and awareness about the harmful effects of electronic cigarettes. The health system and professional orders should develop information and awareness tools to help oral health professionals in their interventions with e-cigarette users.

## Figures and Tables

**Figure 2:**
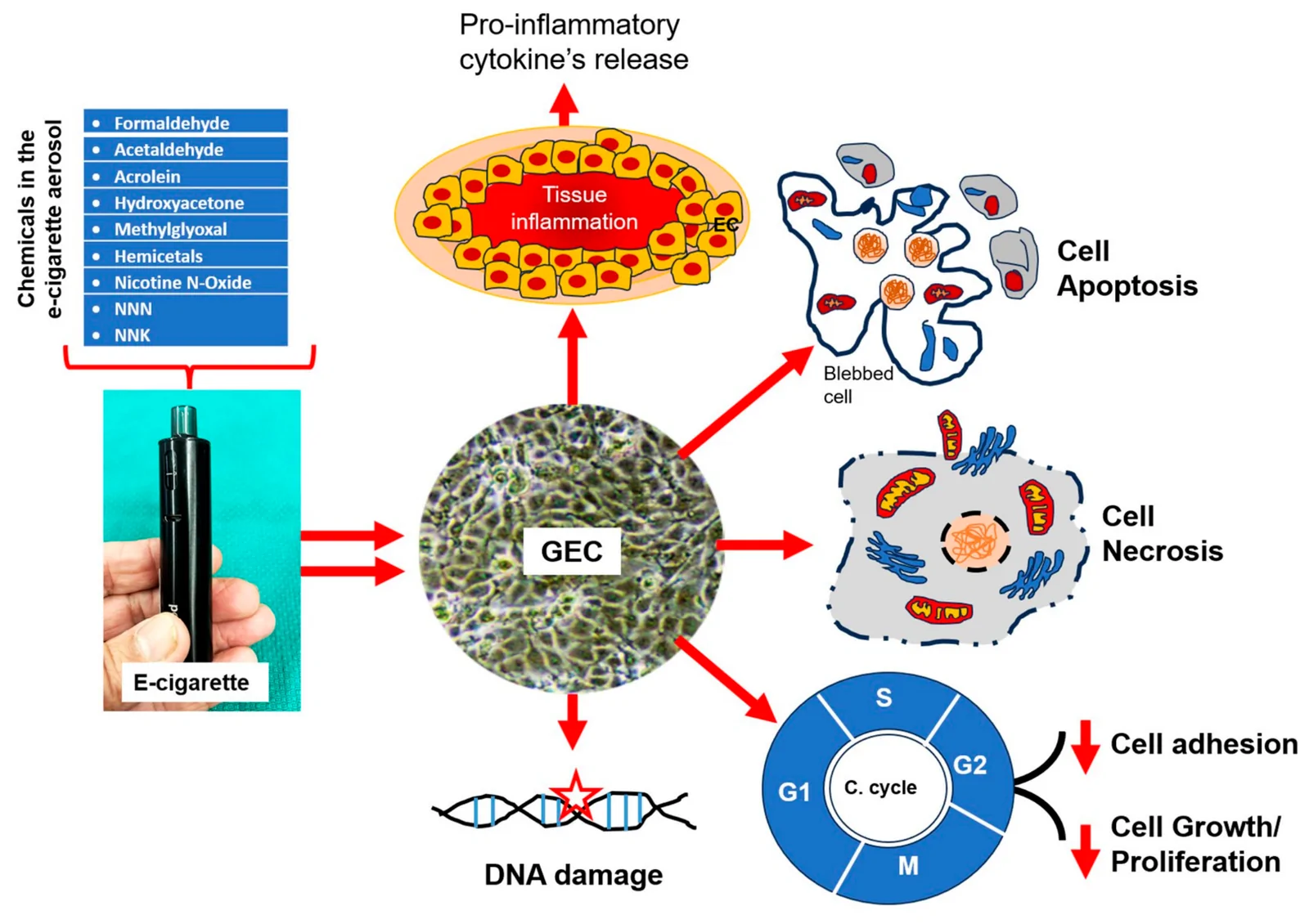
Specific effects of electronic cigarette aerosols on oral cell behavior. Chemicals in electronic cigarette aerosols promote oral cell apoptosis/necrosis, reduce cell viability/growth, increase proinflammatory cytokine secretion, and cause DNA damage. The figure was designed by the author. (GEC), gingival epithelila cells; (M) mitosis; (G), Gap; (S) s-phase; (↓), decrease.

**Figure 3:**
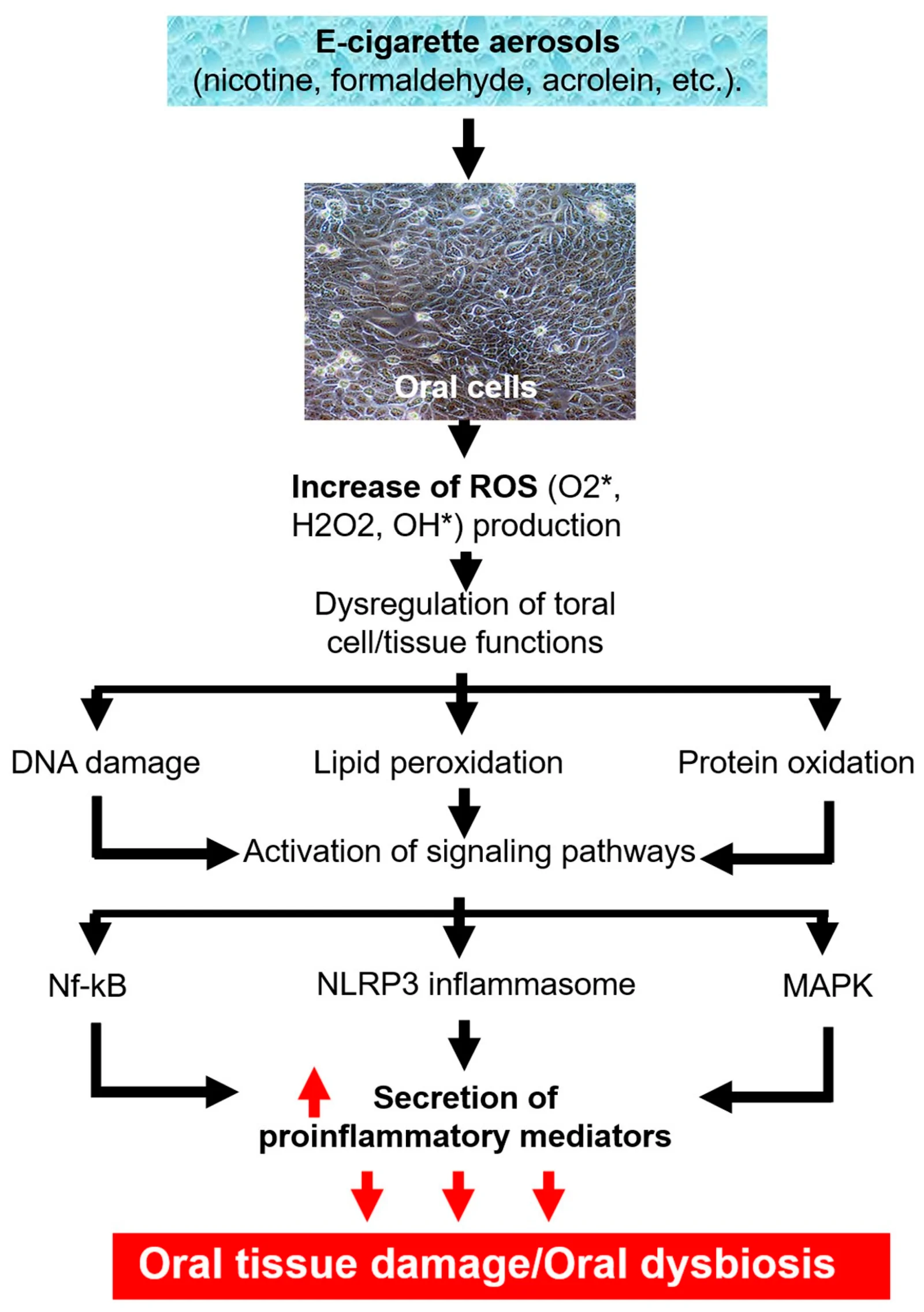
E-cigarette aerosol increases reactive oxygen species (ROS) production and triggers cellular and molecular consequences in oral tissues. Exposure of oral cells to e-cigarette aerosols increases intracellular ROS production through mitochondrial dysfunction and activation of NADPH oxidases (NOX). ROSs cause oxidative stress that leads to DNA damage, lipid peroxidation, and oxidation of cellular proteins. These alterations activate several redox-sensitive signaling pathways, including NF-κB, MAPK, and the NLRP3 inflammasome, leading to high levels of proinflammatory mediators and oral dysbiosis. Collectively, these mechanisms lead to oral diseases.

**Figure 4:**
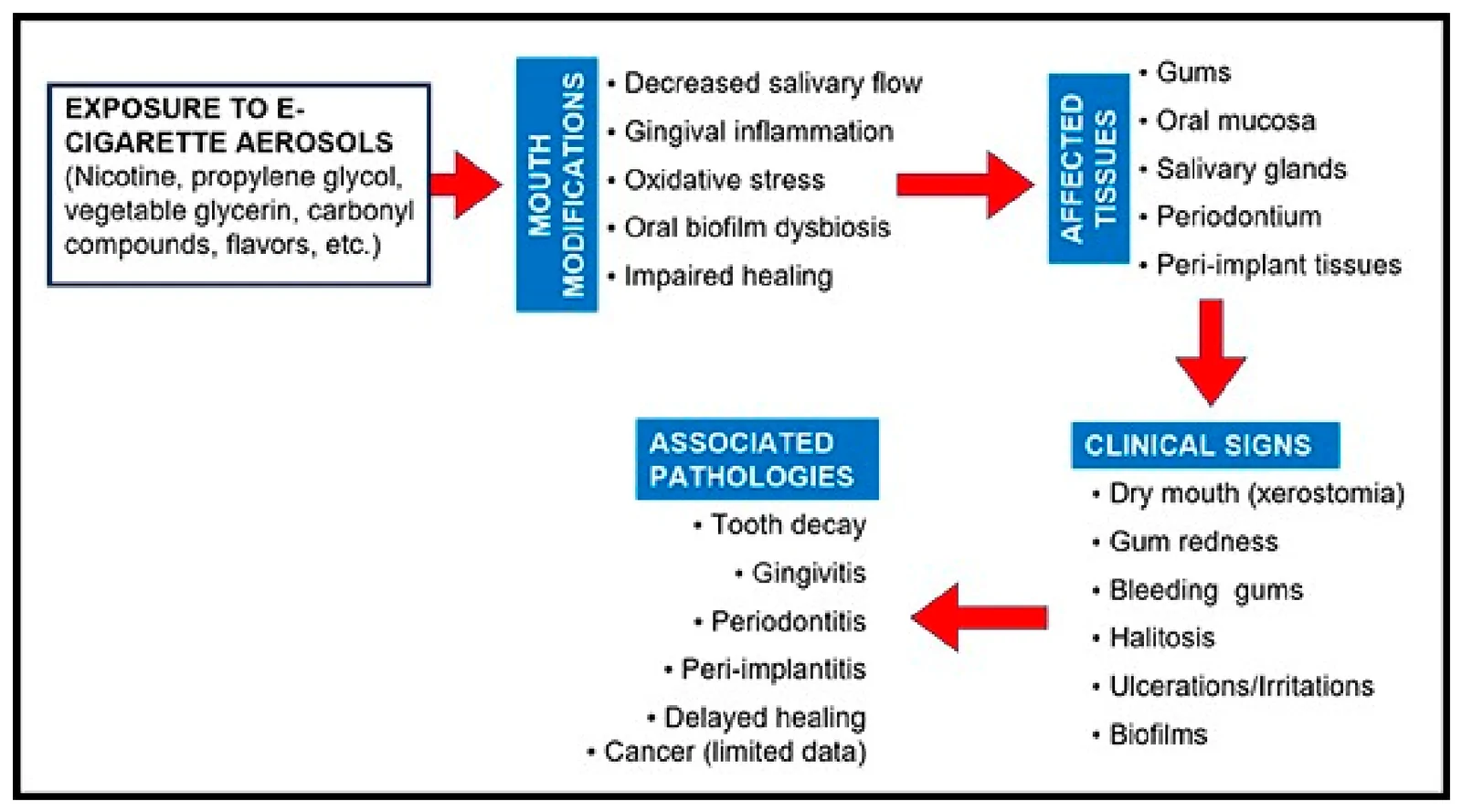
Summary diagram illustrates the relationships between the major chemical components of electronic cigarette aerosols and their proposed effects on oral cells/tissues and disease pathways. The figure was generated using available literature.

**Table 1:** The different generations of electronic cigarettes.

Generation	Design Periods	Device Type	Technical Characteristics
1st generation (cigalike)	2003–2004	Non-rechargeable model	Small size, low-power battery, non-rechargeable model
2nd generation (vape pens)	2009–2010	Rechargeable model	More powerful battery, rechargeable tank, choice of e-liquids
3rd generation (mods)	2013–2014	Large rechargeable and changeable device	Power/voltage adjustment, interchangeable resistors, high aerosol production
4th generation (pods)	2016–2017	Compact systems with replaceable cartridges (pods)	Low-profile design, low power, but high efficiency, nicotine salts

**Table 2:** Temperatures generated by different generations of electronic cigarettes.

Generation	Minimum Observed Temperature (°C)	Maximum Observed Temperature (°C)	References
1st generation	~100–150 °C	~200–250 °C	[85]
2nd generation	~150 °C	~300 °C	[86]
3rd generation	~157 °C	~266 °C	[87,88]
4th generation	~200 °C	>400 °C (up to 430–500 °C locally)	[89,90]
Ceramic Cartridges/THC	~375 °C	~569 °C	[91]

**Table 3:** Chemicals generated by thermal degradation of PG during vaping.

Electronic Cigarette Generation	Temperature of the Resistor (°C)	Chemicals Formed from the PG	Scientific References
1st generation: cigalikes, cartomizers	<5 W ≈100–200 °C (Wet conditions)	Formaldehyde, acetaldehyde (small quantities), formic acid, and acrylic	[85]
2nd generation eGo, clearomiseurs	5–10 W ≈150–300 °C (Wet-partly dry)	Formaldehyde, acetaldehyde, acetone, propionaldehyde	[86,109]
3rd generation Adjustable Mods (Wattage)	20–100 W ≈200–350 °C	Sharp increase: formaldehyde, acetaldehyde, acrolein, methylglyoxal, ethanediol	[87,111]
4th generation Temperature Control Mods (TC)	6–15 W ≈180–250 °C (theoretical, variable)	Formaldehyde and acetaldehyde are still detected; partial reduction if TC is effective	[89,112]
Extreme use/dry hit Dry wick, strong power	>350–1000 °C (locally)	Massive spikes in formaldehyde, acetaldehyde, acrolein; Multiple carbonyl compounds	[86,109]

**Table 5:** Chemical generated after electronic cigarette heating of nicotine-rich e-liquid.

Electronic Cigarette Generation	Power/Typical Temperature	Main Toxic Compounds Generated	References
1st generation: Cigalikes, cartomizers	<5 W ≈100–200 °C (wet conditions)	Low levels of formaldehyde, acetaldehyde	[128]
2nd generation eGo, clearomizers	5–10 W ≈150–300 °C (wet to partially dry)	Formaldehyde, acetaldehyde, acrolein	[107,129]
3rd Generation Adjustable Mods (Wattage)	20–100 W ≈200–350 °C	Hemiacetal, formaldehyde, acrolein, ethanediol	[130,131]
4th Generation Temperature Control (TC) Mods	6–15 W ≈180–250 °C	Lower overall levels of carbonyls, but dependent on the liquid	[112]
Extreme use/dry hit: dry wick, strong power	>350–1000 °C	Thermal aldehyde reduction	[109,132]

**Table 6:** Chemicals being generated by heating flavors while vaping.

**Electronic Cigarette Generation**	**Power/Temperature**	**Flavors**	**Chemicals Formed After Heating**	**References**
1st generation: Cigalikes, cartomizers	<5 W ≈100–200 °C (wet conditions)	Fruity, tobacco	Formaldehyde, acetaldehyde (traces)	[138,146]
2nd generation eGo, clearomizers	5–10 W ≈150–300 °C (wet to partially dry)	Fruity, vanilla, menthol	Formaldehyde, acetaldehyde, acetone	[141,147]
3rd Generation Adjustable Mods (Wattage)	20–100 W ≈200–350 °C	Cinnamon, vanilla, caramel, butter	Benzaldehyde, oxidized vanillin gives vanillic acid, diacetyl, acetylpropionyl, acrolein	[138,148]
4th Generation Temperature Control (TC) Mods	6–15 W ≈180–250 °C	Complex fruity, minty	Benzaldehyde, formaldehyde, secondary carbonyl compounds	[115,135]
Extreme use/dry heat, dry wick, strong power	>350–1000 °C	All flavors	Furan, 5-hydroxymethylfurfural (HMF), phenols, polyfunctional aromatic compounds	[135]

**Table 7:** The main chemical compounds identified in electronic cigarette aerosols.

Chemical Compounds Formed	Main Precursor	References
Formaldehyde	PG, VG	[85,150]
Acetaldehyde	PG, VG	[85,150]
Acrolein	VG	[139,151]
Hydroxyacetone	PG	[139,151,152]
Methylglyoxal	PG, VG	[139,151]
Formaldehyde hemicetals	PG/VG	[150,153,154]
Nicotine N-Oxide	Nicotine	[151,154]
4(methylnitrosamino)-1-(3pyridyl)-1butanone (NNK), N-nitrosonornicotine (NNN)	Nicotine	[155,156,157,158]
Benzaldehyde	Flavors	[159,160]
Diacetyl	Flavors	[161,162]

## Data Availability

No new data were created or analyzed in this study. Data sharing is not applicable to this article.

## Abbreviations

4(methylnitrosamino)-1-(3pyridyl)-1butanone (NNK); aldehyde dehydrogenase (ALDH2); Epidermal growth factor receptor (EGFR); formaldehyde (FA); gingival epithelial cells (GEC); inhibitor of nuclear factor kappa-B kinase (IKK); interleukin-1β (IL-1β); methylglyoxal (MG); mitogen-activated protein kinase (MAPK); nicotinamide adenine dinucleotide phosphate (NADPH); N-nitrosonornicotine (NNN); nuclear factor kappa-B kinase (NF-κB); nucleotide-binding oligomerization domain, Leucine-rich Repeat and Pyrin (NLRP); propylene glycol (PG); reactive oxygen species (ROS); Temperature Control (TC) Mods; vegetable glycerin (VG).

## References

[1] Sharma S., Meister M., Weaver S., Zelikoff J., Bell-Huff C., Black M., Shannahan J., Wright C. (2025). The Beginning of ENDS (Electronic Nicotine Delivery Systems): Origins, Trends, and Regulatory Considerations. Inhal. Toxicol..

[2] Baldassarri S.R. (2020). Electronic Cigarettes: Past, Present, and Future: What Clinicians Need to Know. Clin. Chest Med..

[3] O’Connor R., Schneller L.M., Felicione N.J., Talhout R., Goniewicz M.L., Ashley D.L. (2022). Evolution of Tobacco Products: Recent History and Future Directions. Tob. Control.

[4] Rüther T., Hagedorn D., Schiela K., Schettgen T., Osiander-Fuchs H., Schober W. (2018). Nicotine Delivery Efficiency of First- and Second-Generation Electronic Cigarettes and Its Impact on Relief of Craving during the Acute Phase of Use. Int. J. Hyg. Environ. Health.

[5] Williams M., Talbot P. (2019). Design Features in Multiple Generations of Electronic Cigarette Atomizers. Int. J. Environ. Res. Public Health.

[6] Hammond D., Reid J.L., Rynard V.L., Fong G.T., Cummings K.M., McNeill A., Hitchman S., Thrasher J.F., Goniewicz M.L., Bansal-Travers M. (2019). Prevalence of Vaping and Smoking among Adolescents in Canada, England, and the United States: Repeat National Cross Sectional Surveys. BMJ.

[7] Carpenter M.J., Smith T.T., Wahlquist A.E., Toll B.A., Cropsey K.L., Ware E.C., Kaczmar J.M., Dahne J., Gray K.M. (2026). Medication Samples and Smoking Cessation Among Adults: A Randomized Clinical Trial. JAMA Netw. Open.

[8] Aksoy H.B. (2026). Real-World Effectiveness of Cytisine, Bupropion, and Nicotine Replacement Therapy in a Smoking Cessation Clinic: A Single-Center Retrospective Cohort Study. Tob. Induc. Dis..

[9] Hameed A., Malik D. (2024). Clinical Study Protocol on Electronic Cigarettes and Nicotine Pouches for Smoking Cessation in Pakistan: A Randomized Controlled Trial. Trials.

[10] Xie L., Yan Y., Yang Y., Nan Y., Xiao L. (2023). Evaluation of Smoking Cessation Intervention Effectiveness in Smoking Cessation Clinics-China, 2019–2021. China CDC Wkly..

[11] Hajek P., Phillips-Waller A., Przulj D., Pesola F., Myers Smith K., Bisal N., Li J., Parrott S., Sasieni P., Dawkins L. (2019). A Randomized Trial of Electronic Cigarettes versus Nicotine-Replacement Therapy. N. Engl. J. Med..

[12] Volpp K.G., Troxel A.B., Pauly M.V., Glick H.A., Puig A., Asch D.A., Galvin R., Zhu J., Wan F., DeGuzman J. (2009). A Randomized, Controlled Trial of Financial Incentives for Smoking Cessation. N. Engl. J. Med..

[13] Lu Y., Morimoto K. (2008). Exposure Level to Cigarette Tar or Nicotine Is Associated with Leukocyte DNA Damage in Male Japanese Smokers. Mutagenesis.

[14] Heide J., Adam T.W., Jacobs E., Wolter J.M., Ehlert S., Walte A., Zimmermann R. (2021). Puff-Resolved Analysis and Selected Quantification of Chemicals in the Gas Phase of Electronic Cigarettes, Heat-Not-Burn Devices, and Conventional Cigarettes Using Single-Photon Ionization Time-of-Flight Mass Spectrometry (SPI-TOFMS): A Comparative Study. Nicotine Tob. Res..

[15] Sadasivam R.S., Stevens E.M., Wijesundara J., Wang B., Balakrishnan K., Najjar R., Frisard C., Ballambat N., Pbert L. (2026). Feasibility and Engagement of a Peer-Driven Mobile Intervention for Adolescent Electronic Cigarette Cessation: Cluster Randomized Pilot Study. JMIR Form. Res..

[16] Kirchner T.R., Tian D., Li J., Srivastava P., Zheng Y. (2024). Cigarette Smoking, Electronic Cigarette Use, and Sociodemographic Correlates of Mental Health and Tobacco-Related Disease Risk in the All of Us Research Program. J. Am. Med. Inform. Assoc..

[17] Akinkugbe A.A. (2019). Cigarettes, Electronic Cigarettes, and Adolescents’ Oral Health: Findings from the Population Assessment of Tobacco and Health (PATH) Study. JDR Clin. Trans. Res..

[18] ArRejaie A.S., Al-Aali K.A., Alrabiah M., Vohra F., Mokeem S.A., Basunbul G., Alrahlah A., Abduljabbar T. (2019). Proinflammatory Cytokine Levels and Peri-Implant Parameters among Cigarette Smokers, Individuals Vaping Electronic Cigarettes, and Non-Smokers. J. Periodontol..

[19] Cichońska D., Kusiak A., Kochańska B., Ochocińska J., Świetlik D. (2022). Influence of Electronic Cigarettes on Selected Physicochemical Properties of Saliva. Int. J. Environ. Res. Public Health.

[20] Cichońska D., Król O., Słomińska E.M., Kochańska B., Świetlik D., Ochocińska J., Kusiak A. (2021). Influence of Electronic Cigarettes on Antioxidant Capacity and Nucleotide Metabolites in Saliva. Toxics.

[21] Nigar S., Hassan S., Maqsood A., Ahmed N., Al-Askar M., Mokeem S.A., Alqutub M.N., Aldahian N., Alsagob E.I., Almubarak A.A. (2022). An Assessment of Unstimulated Salivary Flow Rate, IgA and Clinical Oral Dryness among Active and Passive Smokers. Int. J. Occup. Med. Environ. Health.

[22] King J.L., Reboussin B.A., Wiseman K.D., Ribisl K.M., Seidenberg A.B., Wagoner K.G., Wolfson M., Sutfin E.L. (2019). Adverse Symptoms Users Attribute to Electronic Cigarettes: Results from a National Survey of US Adults. Drug Alcohol Depend..

[23] Alsenani M., Alothman L., Alsenaidi J., Alnowaiser A., Aldawsari M., Alharbi N. (2025). Correlation between E-Cigarette Use and Salivary Flow Rate, pH, and Buffering Capacity: A Cross-Sectional Pilot Study. BMC Oral Health.

[24] Tashkandi M., Nourah D., Alhazmi H., Alsaleh O., Almaghraby M., Alzahrani A., Al-Qurashi A., Saeed A., Alasmari R. (2025). Comparative Assessment of Oral Health Findings among Electronic Cigarette Users, Conventional Smokers, and Non-Smokers. Open Dent. J..

[25] Wang X., Mi Q., Yang J., Guan Y., Zeng W., Xiang H., Liu X., Yang W., Yang G., Li X. (2022). Effect of Electronic Cigarette and Tobacco Smoking on the Human Saliva Microbial Community. Braz. J. Microbiol..

[26] Zięba S., Błachnio-Zabielska A., Maciejczyk M., Pogodzińska K., Szuta M., Lo Giudice G., Lo Giudice R., Zalewska A. (2024). Impact of Smoking on Salivary Lipid Profile and Oxidative Stress in Young Adults: A Comparative Analysis between Traditional Cigarettes, E-Cigarettes, and Heat-Not-Burn Products. Med. Sci. Monit..

[27] Majid O.W. (2024). Salivary Lipid Changes in Young Adult Tobacco Smokers and E-Cigarette Users: A Hidden Risk to Oral Health?. Evid.-Based Dent..

[28] Carvalho B.F.D.C., Faria N.C., Silva K.C.S., Greenfield E., Alves M.G.O., Dias M., Mendes M.A., Pérez-Sayáns M., Almeida J.D. (2024). Salivary Metabolic Pathway Alterations in Brazilian E-Cigarette Users. Int. J. Mol. Sci..

[29] Hasan N.W.M., Baharin B., Mohd N., Rahman M.A., Hassan N. (2024). Comparative Effects of E-Cigarette Smoking on Periodontal Status, Salivary pH, and Cotinine Levels. BMC Oral Health.

[30] Kamal N.M., Shams N.S. (2022). The Impact of Tobacco Smoking and Electronic Cigarette Vaping on Salivary Biomarkers: A Comparative Study. Saudi Dent. J..

[31] Pushalkar S., Paul B., Li Q., Yang J., Vasconcelos R., Makwana S., González J.M., Shah S., Xie C., Janal M.N. (2020). Electronic Cigarette Aerosol Modulates the Oral Microbiome and Increases Risk of Infection. iScience.

[32] Ganesan S.M., Dabdoub S.M., Nagaraja H.N., Scott M.L., Pamulapati S., Berman M.L., Shields P.G., Wewers M.E., Kumar P.S. (2020). Adverse Effects of Electronic Cigarettes on the Disease-Naive Oral Microbiome. Sci. Adv..

[33] Kurniawan A.V., Amtha R., Gunardi I., Heriandi A., Sari E.F. (2025). The Impact of Electronic and Conventional Cigarette Use towards Saliva Profile and Oral Microbiota in Adolescents. Asian Pac. J. Cancer Prev..

[34] Rouabhia M., Semlali A. (2021). Electronic Cigarette Vapor Increases *Streptococcus mutans* Growth, Adhesion, Biofilm Formation, and Expression of the Biofilm-Associated Genes. Oral Dis..

[35] Mainardes C.C., Amri O., Palomari Spolidorio M.D., Rouabhia M. (2026). Cannabidiol (CBD) Inhibits *Streptococcus oralis* Growth and Biofilm Formation, while Maintaining Human Gingival Epithelial Cell Viability: An In Vitro Study. Int. J. Dent..

[36] Kulshrestha A., Gupta P. (2023). Combating Polymicrobial Biofilm: Recent Approaches. Folia Microbiol..

[37] Souza-Gabriel A.E., Paschoini-Costa V.L., Sousa-Neto M.D., Silva-Sousa A.C. (2025). Electronic Cigarette Exposure Increases Caries Risk and Modifies Dental Surface in an In Vitro Model. Arch. Oral Biol..

[38] Bepu D.A.N., Silva-Sousa A.C., Lopes-Olhê F.C., Guedes D.F.C., Faria-e-Silva A.L., Mazzi-Chaves J.F., Sousa-Neto M.D., Souza-Gabriel A.E. (2026). Experimental In Vitro Simulation of the Impact of Electronic Cigarette Vapors on Enamel and Dentin. Odontology.

[39] Fairchild R., Setarehnejad A. (2021). Erosive potential of commonly available vapes: A cause for concern?. Br. Dent. J..

[40] Pintado-Palomino K., de Almeida C.V.V.B., Oliveira-Santos C., Pires-de-Souza F.P., Tirapelli C. (2019). The Effect of Electronic Cigarettes on Dental Enamel Color. J. Esthet. Restor. Dent..

[41] Ibrahim M.S., Alatiyyah F.M., Alsalman A.M., Alzenidi R.F., Albattat A.A., Alkhaldi A.S. (2025). Nicotine-Induced Changes in Surface Properties of Restorative Materials and Dental Enamel: An In Vitro Study on Flavored Electronic Cigarette Exposure. Tob. Induc. Dis..

[42] Erdogan M.A., Kalyoncuoğlu Ü.T., Yilmaz Erdemli B. (2025). Comparative Effects of Conventional and Electronic Cigarettes on Discoloration and Surface Roughness of Gingiva-Colored Dental Materials. J. Prosthodont..

[43] Vohra F., Andejani A.F., Alamri O., Alshehri A., Al-Hamdan R.S., Almohareb T., Abduljabbar T. (2020). Influence of Electronic Nicotine Delivery Systems (ENDS) in Comparison to Conventional Cigarette on Color Stability of Dental Restorative Materials. Pak. J. Med. Sci..

[44] Abanto-Vásquez A., Ulloa-Cueva T., Portales-Carbonel C., Curo-Valdivia Y. (2025). Influence of Electronic Cigarette Vapour on the In Vitro Chromatic Stability of Different Nanohybrid Composite Resins. Biomater. Investig. Dent..

[45] Alrabeah G., Habib S.R., Alamro N.M., Alzaaqi M.A. (2023). Evaluation of the Effect of Electronic Cigarette Devices/Vape on the Color of Dental Ceramics: An In Vitro Investigation. Materials.

[46] Dalrymple A., Badrock T.C., Terry A., Barber M., Hall P.J., Thorne D., Gaca M.D., Coburn S., Proctor C. (2018). Assessment of Enamel Discoloration In Vitro Following Exposure to Cigarette Smoke and Emissions from Novel Vapor and Tobacco Heating Products. Am. J. Dent..

[47] Atik E., Şanlı B., Erdogan M.A., Erdemli B.Y., Kalyoncuoğlu Ü.T. (2026). Color Stability of Different Aligner Attachment Composites Following Exposure to Conventional and Electronic Cigarette Smoke. Odontology.

[48] Hashimoto H., Shimazaki Y., Nonoyama T., Kito H., Segawa N., Okai M., Uchibori N., Tomaru S., Kawahara M. (2025). Associations among Smoking Status, Including the Use of Cigarettes, Electronic Cigarettes, and Heated Cigarettes, Periodontal Condition, and Tooth Loss in Employed Japanese Adults: A Cross-Sectional Study. BMC Oral Health.

[49] Hmood A.N., Ebaya M.M.A., El-Embaby A.E. (2025). The In Vitro Effect of Electronic versus Conventional Cigarettes on Color Stability and Surface Roughness of Dental Resin Composites. Sci. Rep..

[50] Alnasser H.A., Elhejazi A.A., Al-Abdulaziz A.A., Alajlan S.S., Habib S.R. (2021). Effect of Conventional and Electronic Cigarettes Smoking on the Color Stability and Translucency of Tooth Colored Restorative Materials: An In Vitro Analysis. Coatings.

[51] Zanetti F., Zhao X., Pan J., Peitsch M.C., Hoeng J., Ren Y. (2019). Effects of Cigarette Smoke and Tobacco Heating Aerosol on Color Stability of Dental Enamel, Dentin, and Composite Resin Restorations. Quintessence Int..

[52] Alhajj M.N., Al-Maweri S.A., Folayan M.O., Halboub E., Khader Y., Omar R., Amran A.G., Al-Batayneh O.B., Celebić A., Persic S. (2022). Oral Health Practices and Self-Reported Adverse Effects of Electronic Cigarette Use among Dental Students in 11 Countries: An Online Survey. BMC Oral Health.

[53] Cho J.H. (2017). The Association between Electronic-Cigarette Use and Self-Reported Oral Symptoms Including Cracked or Broken Teeth and Tongue and/or Inside-Cheek Pain among Adolescents: A Cross-Sectional Study. PLoS ONE.

[54] Kumar P.S., Beverly M.L., Galindo-Fernandez P., Thompson W., Varma M., Shick E. (2026). To Vape or Not to Vape: Oral Health in the Age of Electronic Nicotine Delivery Systems and Electronic Non-Nicotine Delivery Systems. Int. Dent. J..

[55] Pullicin A.J., Kim H., Brinkman M.C., Buehler S.S., Clark P.I., Lim J. (2020). Impacts of Nicotine and Flavoring on the Sensory Perception of Electronic Cigarette Aerosol. Nicotine Tob. Res..

[56] Tolba Y.M., Omar S.S., El Hak A.R., Nagui D.A. (2023). Electronic Cigarettes Can Damage Lingual Papillae and Taste Buds. Can Vitamins C and E Supplementation Reverse This Damage? Life Sci..

[57] Saleh S.M., Shata A., Tiryag A.M., Malak B.G., Okour A.M.A., Atiyah M.A., Al-Khaboori W., Raslan M., Helmy A.A., Al-Howaity A. (2025). Halitosis among Users of Electronic Nicotine Delivery Systems in a Multi-Center Cross-Sectional Study. Sci. Rep..

[58] Mokeem S.A., Alasqah M.N., Michelogiannakis D., Al-Kheraif A.A., Romanos G.E., Javed F. (2018). Clinical and Radiographic Periodontal Status and Whole Salivary Cotinine, IL-1β and IL-6 Levels in Cigarette- and Waterpipe-Smokers and E-Cig Users. Environ. Toxicol. Pharmacol..

[59] Farinha H., Martins V. (2015). Lingua Villosa Nigra Associated with the Use of Electronic Cigarette. Acta Med. Port..

[60] Pannuti C.M., Silva T.E.D., Aquino S.G., Steffens J.P., Nascimento G.G., Leite F.R.M. (2026). E-Cigarettes and Periodontal Health: Current Evidence and Research Gaps. Braz. Oral Res..

[61] Alanazi H., Park H.J., Chakir J., Semlali A., Rouabhia M. (2018). Comparative Study of the Effects of Cigarette Smoke and Electronic Cigarettes on Human Gingival Fibroblast Proliferation, Migration and Apoptosis. Food Chem. Toxicol..

[62] Jeong W., Choi D.W., Kim Y.K., Lee H.J., Lee S.A., Park E.C., Jang S.I. (2020). Associations of Electronic and Conventional Cigarette Use with Periodontal Disease in South Korean Adults. J. Periodontol..

[63] Alnufaiy B., Alzahrani H.G., Lambarte R.A., Alghamdi M.A., Alali A.S., Alanazi W.F., Alkanhal M.H., Almukhalfi A.F. (2025). The Impact of Electronic Cigarette Smoking on Periodontal Status and Proinflammatory Cytokine Levels: A Cross-Sectional Study. BMC Oral Health.

[64] Alanazi H., Rouabhia M. (2022). Effect of Electronic Cigarette Aerosol on Gingival Mucosa Structure and Proinflammatory Cytokine Response. Toxicol. Rep..

[65] BinShabaib M., AlHarthi S.S., Akram Z., Khan J., Rahman I., Romanos G.E., Javed F. (2019). Clinical Periodontal Status and Gingival Crevicular Fluid Cytokine Profile among Cigarette-Smokers, Electronic-Cigarette Users and Never-Smokers. Arch. Oral Biol..

[66] Soysal F., Oner F., Guney Z., Zarinkamar M.S., Shahsavani K., Serdar M.A., Gokmenoglu C. (2026). Subclinical Oxidative and Matrix-Regulatory Alterations Associated with Cigarette Smoking and Electronic Cigarette Use in Periodontally Healthy Adults: A Cross-Sectional Study. J. Clin. Med..

[67] Aljoghaiman E.A., Albrahim A., Aldarisi A., Alsafwani M., Alhalal F. (2026). The Effect of Cigarette and Electronic Cigarette Use on Periodontal Health: A Cross-Sectional Study in Eastern Province, Saudi Arabia. Tob. Induc. Dis..

[68] Rouabhia M., Park H.J., Semlali A., Zakrzewski A., Chmielewski W., Chakir J. (2017). Electronic Cigarette Vapor Induces an Apoptotic Response in Human Gingival Epithelial Cells Through the Caspase-3 Pathway. J. Cell. Physiol..

[69] Vermehren M.F., Wiesmann N., Deschner J., Brieger J., Al-Nawas B., Kämmerer P.W. (2020). Comparative Analysis of the Impact of E-Cigarette Vapor and Cigarette Smoke on Human Gingival Fibroblasts. Toxicol. Vitr..

[70] Sundar I.K., Javed F., Romanos G.E., Rahman I. (2016). Electronic Cigarettes and Flavorings Induce Inflammatory and Pro-Senescence Responses in Oral Epithelial Cells and Periodontal Fibroblasts. Oncotarget.

[71] El-Mouelhy A.T.M., Nasry S.A., Abou El-Dahab O., Sabry D., Fawzy El-Sayed K. (2022). In Vitro Evaluation of the Effect of the Electronic Cigarette Aerosol, Cannabis Smoke, and Conventional Cigarette Smoke on the Properties of Gingival Fibroblasts/Gingival Mesenchymal Stem Cells. J. Periodontal Res..

[72] Fracol M., Dorfman R., Janes L., Kulkarni S., Bethke K., Hansen N., Kim J. (2017). The Surgical Impact of Electronic Cigarettes: A Case Report and Review of the Current Literature. Arch. Plast. Surg..

[73] Xu F., Aboseria E., Janal M.N., Pushalkar S., Bederoff M.V., Vasconcelos R., Sapru S., Paul B., Queiroz E., Makwana S. (2021). Comparative Effects of Electronic Cigarette Aerosol on Periodontium of Periodontitis Patients. Front. Oral Health.

[74] Bardellini E., Amadori F., Conti G., Majorana A. (2018). Oral mucosal lesions in electronic cigarettes consumers versus former smokers. Acta Odontol. Scand..

[75] Santo A.N.T., Hidayat W., Dewi T.S. (2024). Vaping as a Risk Factor for Oral Erythema Multiforme: A Case Report and Literature Review. Int. Med. Case Rep. J..

[76] Kotha A., Mandapati S.R., Rudraraju H. (2024). Potential Association Between Vaping and Recurrent Aphthous Stomatitis: A Case Series. Cureus.

[77] Vámos O., Kulcsár N., Mikecs B., Kelemen K., Kaán R., Abafalvi L., Dinya E., Vág J., Hermann P., Kispélyi B. (2024). Acute effects of traditional and electronic cigarettes on palatal blood flow in smokers: A cross-over pilot study. J. Oral Biol. Craniofac. Res..

[78] Reeve G.S., Rostami M.R., Reich R.F., Behrman D.A., Leopold P.L., Crystal R.G., Strulovici-Barel Y. (2023). Oral epithelium response of electronic cigarette users to electronic cigarette. J. Oral Pathol. Med..

[79] Sekar G., Gundlapalle P., Elango S., Surendran S., Anitha K., Acchuthakrishnan K.A., Nagappan N. (2025). Vaping Troubles: Exploring the Link Between E-Cigarettes and Oral Lesions in India’s Urban Youth. J. Pharm. Bioallied Sci..

[80] de Lima J.M., Macedo C.C.S., Barbosa G.V., Castellano L.R.C., Hier M.P., Alaoui-Jamali M.A., da Silva S.D. (2023). E-Liquid Alters Oral Epithelial Cell Function to Promote Epithelial to Mesenchymal Transition and Invasiveness in Preclinical Oral Squamous Cell Carcinoma. Sci. Rep..

[81] Pilati S.F.M., Pilati P.V.F. (2023). A New In Vitro Study Demonstrates That Electronic Cigarettes Promote Cellular Carcinogenic Characteristics. Evid.-Based Dent..

[82] Guo J., Hecht S.S. (2023). DNA Damage in Human Oral Cells Induced by Use of Electronic Cigarettes. Drug Test. Anal..

[83] Murphy S. (2023). What Electronic Cigarette Factors Determine Oral Epithelial DNA Damage in Consumers?. Evid.-Based Dent..

[84] George J., Tommasi S., Pabustan N., Kessler D.M., Thomas Z.L., Baezconde-Garbanati L., Siegmund K.D., Besaratinia A. (2026). Multidimensional Exposure Architecture Shapes Vaping-Associated Transcriptomic Dysregulation in Oral Epithelium. Front. Oncol..

[85] Jaegers N.R., Hu W., Weber T.J., Hu J.Z. (2021). Low-Temperature (<200 °C) Degradation of Electronic Nicotine Delivery System Liquids Generates Toxic Aldehydes. Sci. Rep..

[86] Chen W., Wang P., Ito K., Fowles J., Shusterman D., Jaques P.A., Kumagai K. (2018). Measurement of Heating Coil Temperature for Electronic Cigarettes with a “Top-Coil” Clearomizer. PLoS ONE.

[87] Li Y., Burns A.E., Tran L.N., Abellar K.A., Poindexter M., Li X., Madl A.K., Pinkerton K.E., Nguyen T.B. (2021). Impact of E-Liquid Composition, Coil Temperature, and Puff Topography on the Aerosol Chemistry of Electronic Cigarettes. Chem. Res. Toxicol..

[88] Poindexter M.E., Li Y., Madl A.K., Nguyen T.B., Pinkerton K.E. (2025). Increasing Coil Temperature of a Third-Generation Electronic Cigarette Device Modulates C57BL/6 Mouse Lung Immune Cell Composition and Cytokine Milieu Independently of Aerosol Dose. J. Toxicol. Environ. Health A.

[89] Dibaji S.A.R., Guha S., Arab A., Murray B.T., Myers M.R. (2018). Accuracy of Commercial Electronic Nicotine Delivery Systems (ENDS) Temperature Control Technology. PLoS ONE.

[90] Tran L.N., Chiu E.Y., Hunsaker H.C., Wu K.C., Poulin B.A., Madl A.K., Pinkerton K.E., Nguyen T.B. (2023). Carbonyls and Aerosol Mass Generation from Vaping Nicotine Salt Solutions Using Fourth- and Third-Generation Electronic Cigarette Devices: Effects of Coil Resistance, Coil Age, and Coil Metal Material. Chem. Res. Toxicol..

[91] Lynch J., Lorenz L., Brueggemeyer J.L., Lanzarotta A., Falconer T.M., Wilson R.A. (2021). Simultaneous Temperature Measurements and Aerosol Collection During Vaping for the Analysis of Δ^9^-Tetrahydrocannabinol and Vitamin E Acetate Mixtures in Ceramic Coil Style Cartridges. Front. Chem..

[92] Vázquez-López N.I., Falfán-Valencia R., Pérez-Rubio G. (2026). Findings on Electronic Cigarette Flavors and Their Implications for Human Health: A Systematic Review. Front. Public Health.

[93] Zhu S.H., Sun J.Y., Bonnevie E., Cummins S.E., Gamst A., Yin L., Lee M. (2014). Four Hundred and Sixty Brands of E-Cigarettes and Counting: Implications for Product Regulation. Tob. Control.

[94] Hsu G., Sun J.Y., Zhu S.H. (2018). Evolution of Electronic Cigarette Brands From 2013-2014 to 2016-2017: Analysis of Brand Websites. J. Med. Internet Res..

[95] Canada Gazette, Part I, Volume 155, Number 25: Order Amending Schedules 2 and 3 to the Tobacco and Vaping Products Act (Flavours). https://gazette.gc.ca/rp-pr/p1/2021/2021-06-19/html/reg2-eng.html.

[96] FDA (2020). Finalized Enforcement Policy on Flavored Cartridge-Based E-Cigarettes, 2 January 2020; FDA’s Enforcement Priorities for ENDS. https://digitalmedia.hhs.gov.

[97] Directive 2014/40/UE du Parlement Européen et du Conseil du 3 Avril 2014 Relative aux Produits du Tabac; Commission EUROPÉENNE. Electronic Cigarettes—Article 20 of the Tobacco Products Directive. https://eur-lex.europa.eu/eli/dir/2014/40/2023-10-23.

[98] Christen S.E., Hermann L., Bekka E., Vonwyl C., Hammann F., van der Velpen V., Eap C.B., Benowitz N.L., Haschke M., Liakoni E. (2024). Pharmacokinetics and Pharmacodynamics of Inhaled Nicotine Salt and Free-Base Using an E-Cigarette: A Randomized Crossover Study. Nicotine Tob. Res..

[99] Dagla I., Gikas E., Tsarbopoulos A. (2023). Two Fast GC-MS Methods for the Measurement of Nicotine, Propylene Glycol, Vegetable Glycol, Ethylmaltol, Diacetyl, and Acetylpropionyl in Refill Liquids for Electronic Cigarettes. Molecules.

[100] Srbinoska M., Kavrakovski Z., Rafajlovska V., Simonovska J. (2019). Determined and Declared Nicotine Content in Refill Liquids for Electronic Cigarettes Marketed in North Macedonia. Arh. Hig. Rada Toksikol..

[101] Heide M., Engelhard C. (2023). Chemical Analysis of Electronic Cigarette Liquids (E-Liquids) and Direct Nicotine Quantitation Using Surface-Assisted Flowing Atmospheric-Pressure Afterglow Desorption/Ionization Mass Spectrometry (SA-FAPA-MS). RSC Adv..

[102] Bansal M., Sharma M., Bullen C., Svirskis D. (2018). A Stability Indicating HPLC Method to Determine Actual Content and Stability of Nicotine within Electronic Cigarette Liquids. Int. J. Environ. Res. Public Health.

[103] Okolie J.A. (2022). Insights on Production Mechanism and Industrial Applications of Renewable Propylene Glycol. iScience.

[104] Bentefour E.H., Glorieux C., Chirtoc M., Thoen J. (2004). Thermal Relaxation of Glycerol and Propylene Glycol Studied by Photothermal Spectroscopy. J. Chem. Phys..

[105] Laino T., Tuma C., Moor P., Martin E., Stolz S., Curioni A. (2012). Mechanisms of Propylene Glycol and Triacetin Pyrolysis. J. Phys. Chem. A.

[106] Lorkiewicz P., Keith R., Lynch J., Jin L., Theis W., Krivokhizhina T., Riggs D., Bhatnagar A., Srivastava S., Conklin D.J. (2022). Electronic Cigarette Solvents, JUUL E-Liquids, and Biomarkers of Exposure: In Vivo Evidence for Acrolein and Glycidol in E-Cig-Derived Aerosols. Chem. Res. Toxicol..

[107] Kosmider L., Sobczak A., Fik M., Knysak J., Zaciera M., Kurek J., Goniewicz M.L. (2014). Carbonyl Compounds in Electronic Cigarette Vapors: Effects of Nicotine Solvent and Battery Output Voltage. Nicotine Tob. Res..

[108] Uchiyama S., Noguchi M., Sato A., Ishitsuka M., Inaba Y., Kunugita N. (2020). Determination of Thermal Decomposition Products Generated from Electronic Cigarettes. Chem. Res. Toxicol..

[109] Gillman I.G., Pennington A.S.C., Humphries K.E., Oldham M.J. (2020). Determining the Impact of Flavored E-Liquids on Aldehyde Production during Vaping. Regul. Toxicol. Pharmacol..

[110] Saliba N.A., El Hellani A., Honein E., Salman R., Talih S., Zeaiter J., Shihadeh A. (2018). Surface Chemistry of Electronic Cigarette Electrical Heating Coils: Effects of Metal Type on Propylene Glycol Thermal Decomposition. J. Anal. Appl. Pyrolysis.

[111] Sleiman M., Logue J.M., Montesinos V.N., Russell M.L., Litter M.I., Gundel L.A., Destaillats H. (2016). Emissions from Electronic Cigarettes: Key Parameters Affecting the Release of Harmful Chemicals. Environ. Sci. Technol..

[112] Talih S., Salman R., El-Hage R., Karam E., Karaoghlanian N., El-Hellani A., Saliba N., Shihadeh A. (2019). Characteristics and Toxicant Emissions of JUUL Electronic Cigarettes. Tob. Control.

[113] Li J., Tu Y., He K., Chen C., Liang L., Ruan C., Zhang Q. (2025). Mechanistic Insights into Glycerol Oxidation to High-Value Chemicals via Metal-Based Catalysts. Molecules.

[114] Ebersole J., Samburova V., Son Y., Cappelli D., Demopoulos C., Capurro A., Pinto A., Chrzan B., Kingsley K., Howard K. (2020). Harmful Chemicals Emitted from Electronic Cigarettes and Potential Deleterious Effects in the Oral Cavity. Tob. Induc. Dis..

[115] Oldham M.J., Jeong L., Gillman I.G. (2024). An Approach to Flavor Chemical Thermal Degradation Analysis. Toxics.

[116] Strongin R.M., Sharma E., Erythropel H.C., Kassem N.O.F., Noël A., Peyton D.H., Rahman I. (2025). Chemical and Physiological Interactions between E-Liquid Constituents: Cause for Concern?. Tob. Control.

[117] Cecilia J.A., García-Sancho C., Jiménez-Gómez C.P., Moreno-Tost R., Maireles-Torres P. (2018). Porous Silicon-Based Catalysts for the Dehydration of Glycerol to High Value-Added Products. Materials.

[118] Uchiyama S., Ohta K., Inaba Y., Kunugita N. (2013). Determination of Carbonyl Compounds Generated from the Electronic Cigarette Using Coupled Silica Cartridges Impregnated with Hydroquinone and 2,4-Dinitrophenylhydrazine, Followed by High-Performance Liquid Chromatography. Anal. Sci..

[119] Huang J., Guo Y., Lin J., Xia T., Wang L., Mu H., Liu R., Jin Q., Song Z., Wang X. (2022). Effects of Temperature and Ferric Ion on the Formation of Glycerol Core Aldehydes during Simulated Frying. Food Chem..

[120] Jensen R.P., Luo W., Pankow J.F., Strongin R.M., Peyton D.H. (2015). Hidden Formaldehyde in Electronic Cigarette Aerosols. N. Engl. J. Med..

[121] Benowitz N.L. (2009). Pharmacology of Nicotine: Addiction, Smoking-Induced Disease, and Therapeutics. Annu. Rev. Pharmacol. Toxicol..

[122] Angevine D.J., Camacho K.J., Rzayev J., Benedict J.B. (2022). Enhancing the Photo and Thermal Stability of Nicotine through Crystal Engineering with Gentisic Acid. Molecules.

[123] Chavarrio Cañas J.E., Monge-Palacios M., Grajales-González E., Sarathy S.M. (2021). Early Chemistry of Nicotine Degradation in Heat-Not-Burn Smoking Devices and Conventional Cigarettes: Implications for Users and Second- and Third-Hand Smokers. J. Phys. Chem. A.

[124] Eshraghian E.A., Al-Delaimy W.K. (2021). A Review of Constituents Identified in Electronic Cigarette Liquids and Aerosols. Tob. Prev. Cessat..

[125] Yang J., Li Y., Liu C., Tang S., Li Z., Jiang L. (2023). Thermal Properties and Kinetic Analysis of Pyrolysis Products of Nicotine Salts from Electronic Cigarettes Using Pyrolysis Gas Chromatography/Mass Spectrometry. Front. Environ. Sci..

[126] Lee T., Park J., Kim Y., Chen W.H., Kwon E.E. (2025). Comparison of Toxic Pyrogenic Compounds Derived from Conventional Cigarettes and Heated Tobacco Products. J. Hazard. Mater..

[127] Moldoveanu S.C. (2022). Interconversion of nicotine enantiomers during heating and implications for smoke from combustible cigarettes, heated tobacco products, and electronic cigarettes. Chirality.

[128] Goniewicz M.L., Knysak J., Gawron M., Kosmider L., Sobczak A., Kurek J., Prokopowicz A., Jablonska-Czapla M., Rosik-Dulewska C., Havel C. (2014). Levels of Selected Carcinogens and Toxicants in Vapour from Electronic Cigarettes. Tob. Control.

[129] Sala C., Medana C., Pellegrino R., Aigotti R., Bello F.D., Bianchi G., Davoli E. (2017). Dynamic Measurement of Newly Formed Carbonyl Compounds in Vapors from Electronic Cigarettes. Eur. J. Mass Spectrom..

[130] Saleh Q.M., Hensel E.C., Eddingsaas N.C., Robinson R.J. (2021). Effects of Manufacturing Variation in Electronic Cigarette Coil Resistance and Initial Pod Mass on Coil Lifetime and Aerosol Generation. Int. J. Environ. Res. Public Health.

[131] Farsalinos K.E., Voudris V., Poulas K. (2015). Electronic Cigarettes Generate High Levels of Aldehydes Only in ‘Dry Puff’ Conditions. Addiction.

[132] Farsalinos K.E., Gillman G., Poulas K., Voudris V. (2015). Tobacco-Specific Nitrosamines in Electronic Cigarettes: Comparison between Liquid and Aerosol Levels. Int. J. Environ. Res. Public Health.

[133] Barhdadi S., Rogiers V., Deconinck E., Vanhaecke T. (2021). Toxicity Assessment of Flavour Chemicals Used in Electronic Cigarettes: Current State and Future Challenges. Arch. Toxicol..

[134] Leigh N.J., Lawton R.I., Hershberger P.A., Goniewicz M.L. (2016). Flavourings Significantly Affect Inhalation Toxicity of Aerosol Generated from Electronic Nicotine Delivery Systems (ENDS). Tob. Control.

[135] Kuehl P.J., McDonald J.D., Weber D.T., Khlystov A., Nystoriak M.A., Conklin D.J. (2022). Composition of Aerosols from Thermal Degradation of Flavors Used in ENDS and Tobacco Products. Inhal. Toxicol..

[136] Jabba S.V., Diaz A.N., Erythropel H.C., Zimmerman J.B., Jordt S.E. (2020). Chemical Adducts of Reactive Flavor Aldehydes Formed in Electronic Cigarette Liquids Are Cytotoxic and Inhibit Mitochondrial Function in Respiratory Epithelial Cells. Nicotine Tob. Res..

[137] Conklin D.J., Ogunwale M.A., Chen Y., Theis W.S., Nantz M.H., Fu X.A., Chen L.C., Riggs D.W., Lorkiewicz P., Bhatnagar A. (2018). Electronic Cigarette-Generated Aldehydes: The Contribution of E-Liquid Components to Their Formation and the Use of Urinary Aldehyde Metabolites as Biomarkers of Exposure. Aerosol Sci. Technol..

[138] Khlystov A., Samburova V. (2016). Flavoring Compounds Dominate Toxic Aldehyde Production during Electronic Cigarette Vaping. Environ. Sci. Technol..

[139] Fazeli E., Martinez B., Son Y., Khlystov A. (2026). Flavoring Compound Chemical Class and Vaping Conditions Determine Toxic Carbonyl Emissions from Electronic Cigarettes. Chem. Res. Toxicol..

[140] Kishimoto A., Wu D., O’Shea D.F. (2024). Forecasting Vaping Health Risks through Neural Network Model Prediction of Flavour Pyrolysis Reactions. Sci. Rep..

[141] McAdam K., Waters G., Moldoveanu S., Margham J., Cunningham A., Vas C., Porter A., Digard H. (2021). Diacetyl and Other Ketones in Electronic Cigarette Aerosols: Some Important Sources and Contributing Factors. Front. Chem..

[142] Taskingul A.B., Kiran S., Emerce E. (2024). Comparison of Health Complaints, Occupational Risks, and Occupational Health Practices of Healthcare Workers According to Professions and Departments in the Hospital. Cureus.

[143] Bitzer Z.T., Goel R., Reilly S.M., Elias R.J., Silakov A., Foulds J., Muscat J., Richie J.P. (2018). Effect of Flavoring Chemicals on Free Radical Formation in Electronic Cigarette Aerosols. Free Radic. Biol. Med..

[144] Yu X., Zeng X., Xiao F., Chen R., Sinharoy P., Gross E.R. (2022). Electronic Cigarette Aerosol Exacerbates Cardiovascular Oxidative Stress in Mice with an Inactive Aldehyde Dehydrogenase 2 Enzyme. Redox Biol..

[145] Erythropel H.C., Jabba S.V., DeWinter T.M., Mendizabal M., Anastas P.T., Jordt S.E., Zimmerman J.B. (2019). Formation of Flavorant-Propylene Glycol Adducts with Novel Toxicological Properties in Chemically Unstable Electronic Cigarette Liquids. Nicotine Tob. Res..

[146] Kopa-Stojak P.N., Pawliczak R. (2025). Disposable Electronic Cigarettes: Chemical Composition and Health Effects of Their Use. A Systematic Review. Toxicol. Mech. Methods.

[147] Allen J.G., Flanigan S.S., LeBlanc M., Vallarino J., MacNaughton P., Stewart J.H., Christiani D.C. (2016). Flavoring Chemicals in Electronic Cigarettes: Diacetyl, 2,3-Pentanedione, and Acetoin in a Sample of 51 Products, Including Fruit-, Candy-, and Cocktail-Flavored Electronic Cigarettes. Environ. Health Perspect..

[148] Pankow J.F., Kim K., McWhirter K.J., Luo W., Escobedo J.O., Strongin R.M., Duell A.K., Peyton D.H. (2017). Benzene Formation in Electronic Cigarettes. PLoS ONE.

[149] Cook D.K., Lalonde G., Oldham M.J., Wang J., Bates A., Ullah S., Sulaiman C., Carter K., Jongsma C., Dull G. (2024). A Practical Framework for Novel Electronic Nicotine Delivery System Evaluation: Chemical and Toxicological Characterization of JUUL2 Aerosol and Comparison with Reference Cigarettes. Toxics.

[150] Salamanca J.C., Munhenzva I., Escobedo J.O., Jensen R.P., Shaw A., Campbell R., Luo W., Peyton D.H., Strongin R.M. (2017). Formaldehyde Hemiacetal Sampling, Recovery, and Quantification from Electronic Cigarette Aerosols. Sci. Rep..

[151] Yan B., Zagorevski D., Ilievski V., Kleiman N.J., Re D.B., Navas-Acien A., Hilpert M. (2021). Identification of Newly Formed Toxic Chemicals in Electronic Cigarette Aerosols with Orbitrap Mass Spectrometry and Implications on Electronic Cigarette Control. Eur. J. Mass Spectrom..

[152] Deng H., Tang S., Yang F., Chen D., Bian Z., Wang Y., Tang G., Lee H.K. (2023). Recent Advances in the Analysis of Electronic Cigarette Liquids and Aerosols: Sample Preparation and Chromatographic Characterization. J. Chromatogr. A.

[153] Vreeke S., Peyton D.H., Strongin R.M. (2018). Triacetin Enhances Levels of Acrolein, Formaldehyde Hemiacetals, and Acetaldehyde in Electronic Cigarette Aerosols. ACS Omega.

[154] Harris T. (2024). Physical and Chemical Characterization of Aerosols Produced from Experimentally Designed Nicotine Salt-Based E-Liquids. Chem. Res. Toxicol..

[155] Kerber P.J., Duell A.K., Powers M., Strongin R.M., Peyton D.H. (2022). Effects of Common E-Liquid Flavorants and Added Nicotine on Toxicant Formation during Vaping Analyzed by ^1H NMR Spectroscopy. Chem. Res. Toxicol..

[156] Reilly S.M., Cheng T., Feng C., Walters M.J. (2024). Harmful and Potentially Harmful Constituents in E-Liquids and Aerosols from Electronic Nicotine Delivery Systems (ENDS). Chem. Res. Toxicol..

[157] Son Y., Giovenco D.P., Delnevo C., Khlystov A., Samburova V., Meng Q. (2020). Indoor Air Quality and Passive Electronic Cigarette Aerosol Exposures in Vape-Shops. Nicotine Tob. Res..

[158] Kankanamage R.N.T., Ghosh A.B., Jiang D., Gkika K., Keyes T., Achola L.A., Suib S., Rusling J.F. (2020). Metabolites of Tobacco- and Electronic Cigarette-Related Nitrosamines Can Drive Cu^2+^-Mediated DNA Oxidation. Chem. Res. Toxicol..

[159] Klager S., Vallarino J., MacNaughton P., Christiani D.C., Lu Q., Allen J.G. (2017). Flavoring Chemicals and Aldehydes in Electronic Cigarette Emissions. Environ. Sci. Technol..

[160] Martin A., Tempra C., Yu Y., Liekkinen J., Thakker R., Lee H., de Santos Moreno B., Vattulainen I., Rossios C., Javanainen M. (2024). Exposure to Aldehyde Cherry E-Liquid Flavoring and Its Vaping Byproduct Disrupt Pulmonary Surfactant Biophysical Function. Environ. Sci. Technol..

[161] White A.V., Wambui D.W., Pokhrel L.R. (2021). Risk Assessment of Inhaled Diacetyl from Electronic Cigarette Use among Teens and Adults. Sci. Total Environ..

[162] Attfield K.R., Zalay M., Zwack L.M., Glassford E.K., LeBouf R.F., Materna B.L. (2022). Assessment of Worker Chemical Exposures in California Vape Shops. J. Occup. Environ. Hyg..

[163] Valverde-Santiago M., Pontel L.B. (2025). Emerging Mechanisms Underlying Formaldehyde Toxicity and Response. Mol. Cell.

[164] Nadalutti C.A., Prasad R., Wilson S.H. (2021). Perspectives on Formaldehyde Dysregulation: Mitochondrial DNA Damage and Repair in Mammalian Cells. DNA Repair.

[165] Bernardini L., Barbosa E., Charão M.F., Brucker N. (2022). Formaldehyde Toxicity Reports from In Vitro and In Vivo Studies: A Review and Updated Data. Drug Chem. Toxicol..

[166] Rieckher M., Gallrein C., Alquezar-Artieda N., Bourached-Silva N., Vaddavalli P.L., Mares D., Backhaus M., Blindauer T., Greger K., Wiesner E. (2024). Distinct DNA Repair Mechanisms Prevent Formaldehyde Toxicity during Development, Reproduction and Aging. Nucleic Acids Res..

[167] Nilsson J.A., Zheng X., Sundqvist K., Liu Y., Atzori L., Elfwing A., Arvidson K., Grafström R.C. (1998). Toxicity of Formaldehyde to Human Oral Fibroblasts and Epithelial Cells: Influences of Culture Conditions and Role of Thiol Status. J. Dent. Res..

[168] Ma T., Lee A., Eng B., Patel V., Michel S.L.J., Kane M.A., Dalby R., Schneider A. (2025). Aerosolized E-Liquid Base Constituents Induce Cytotoxicity and Genotoxicity in Oral Keratinocytes. Oral Dis..

[169] Said A., Jaedicke C., Ganguly M., Awadalla A., Cottrell J.A. (2026). Chondrocyte Viability and Function is Impaired by E-Cigarette Liquid Flavor Additives. Front. Biosci. (Landmark Ed.).

[170] Tommasi S., Blumenfeld H., Besaratinia A. (2023). Vaping Dose, Device Type, and E-Liquid Flavor are Determinants of DNA Damage in Electronic Cigarette Users. Nicotine Tob. Res..

[171] Khachatoorian C., Luo W., McWhirter K.J., Pankow J.F., Talbot P. (2021). Electronic Cigarette Fluids and Aerosol Residues Cause Oxidative Stress and an Inflammatory Response in Human Keratinocytes and 3D Skin Models. Toxicol. Vitr..

[172] Mourão C.F., Shibli J.A. (2023). What Is the Impact of Electronic Cigarettes on Periodontal Stem Cells as Revealed by Transcriptomic Analyses?. Evid.-Based Dent..

[173] Belkin S., Benthien J., Axt P.N., Mohr T., Mortensen K., Weckmann M., Drömann D., Franzen K.F. (2023). Impact of Heated Tobacco Products, Electronic Cigarettes, and Cigarettes on Inflammation and Endothelial Dysfunction. Int. J. Mol. Sci..

[174] Auschwitz E., Almeda J., Andl C.D. (2023). Mechanisms of Electronic Cigarette Vape-Induced Epithelial Cell Damage. Cells.

[175] Cheng G., Guo J., Carmella S.G., Lindgren B., Ikuemonisan J., Niesen B., Jensen J., Hatsukami D.K., Balbo S., Hecht S.S. (2022). Increased Acrolein-DNA Adducts in Buccal Brushings of Electronic Cigarette Users. Carcinogenesis.

[176] Morgil G.K., Çok İ. (2025). Evaluation and Comparison of DNA Alkylation and Oxidative Damage in Electronic Cigarette and Heated Tobacco Users. Toxicol. Mech. Methods.

[177] Thomas L.A., Hopkinson R.J. (2024). The Biochemistry of the Carcinogenic Alcohol Metabolite Acetaldehyde. DNA Repair.

[178] Sonohara Y., Yamamoto J., Tohashi K., Takatsuka R., Matsuda T., Iwai S., Kuraoka I. (2019). Acetaldehyde Forms Covalent GG Intrastrand Crosslinks in DNA. Sci. Rep..

[179] Vijayraghavan S., Porcher L., Mieczkowski P.A., Saini N. (2022). Acetaldehyde Makes a Distinct Mutation Signature in Single-Stranded DNA. Nucleic Acids Res..

[180] Ogunwale M.A., Li M., Ramakrishnam Raju M.V., Chen Y., Nantz M.H., Conklin D.J., Fu X.A. (2017). Aldehyde Detection in Electronic Cigarette Aerosols. ACS Omega.

[181] Ramenzoni L.L., Schneider A., Fox S.C., Meyer M., Meboldt M., Attin T., Schmidlin P.R. (2022). Cytotoxic and Inflammatory Effects of Electronic and Traditional Cigarettes on Oral Gingival Cells Using a Novel Automated Smoking Instrument: An In Vitro Study. Toxics.

[182] Kobylińska A., Gozdowski D., Studnicki M., Olczak-Kowalczyk D. (2026). Cigarette and Electronic Cigarette Smoking as Associated Factors of Gingivitis in 15-Year-Olds: A Cross-Sectional Study in Poland. Sci. Rep..

[183] Choueiri M.E., Laaboudi F.Z., Zhang Z., Chmielewski W., Rouabhia M. (2025). Evaluation of the Effect of Electronic Cigarette Aerosol on Normal and Diabetic Human Fibroblast Interaction with Dental Implant Materials. Int. J. Oral Maxillofac. Implants.

[184] Yeung C.A. (2023). Is the Use of Tobacco Products, Especially Electronic Nicotine Delivery Systems (ENDS), Associated with the Incidence of Oral Health Outcomes among US Adults?. Evid.-Based Dent..

[185] Tulen C.B.M., Duistermaat E., Cremers J.W.J.M., Klerx W.N.M., Fokkens P.H.B., Weibolt N., Kloosterboer N., Dentener M.A., Gremmer E.R., Jessen P.J.J. (2022). Smoking-Associated Exposure of Human Primary Bronchial Epithelial Cells to Aldehydes: Impact on Molecular Mechanisms Controlling Mitochondrial Content and Function. Cells.

[186] Kassem N.O., Daffa R.M., Liles S., Jackson S.R., Kassem N.O., Younis M.A., Mehta S., Chen M., Jacob P., Carmella S.G. (2014). Children’s Exposure to Secondhand and Thirdhand Smoke Carcinogens and Toxicants in Homes of Hookah Smokers. Nicotine Tob. Res..

[187] Colombo G., Dalle-Donne I., Orioli M., Giustarini D., Rossi R., Clerici M., Regazzoni L., Aldini G., Milzani A., Butterfield D.A. (2012). Oxidative Damage in Human Gingival Fibroblasts Exposed to Cigarette Smoke. Free Radic. Biol. Med..

[188] Álvarez-Martínez E., Porto-Puerta I.E., Ardila C.M. (2025). Genotoxic damage assessment using the micronucleus assay in buccal mucosa of different types of smokers: A cross-sectional study. World J. Exp. Med..

[189] AlHarthi S.S., BinShabaib M., Akram Z., Rahman I., Romanos G.E., Javed F. (2019). Impact of Cigarette Smoking and Vaping on the Outcome of Full-Mouth Ultrasonic Scaling among Patients with Gingival Inflammation: A Prospective Study. Clin. Oral Investig..

[190] Kuntic I., Kuntic M., Oelze M., Stamm P., Karpi A., Kleinert H., Hahad O., Münzel T., Daiber A. (2023). The Role of Acrolein for Electronic Cigarette Vapour Condensate Mediated Activation of NADPH Oxidase in Cultured Endothelial Cells and Macrophages. Pflug. Arch..

[191] Sompa S.I., Upadhyay S., Ganguly K., Bergström A., Ji J., Palmberg L. (2026). Epithelial Effects of Exposure to Electronic Cigarettes Alone or in Combination with Cigarette Smoke Using Multicellular Lung Mucosa Models. Environ. Toxicol. Pharmacol..

[192] Anand N., Emmadi P., Ambalavanan N., Ramakrishnan T. (2011). Effect of a Volatile Smoke Component (Acrolein) on Human Gingival Fibroblasts: An In Vitro Study. J. Indian Soc. Periodontol..

[193] Tsou H.H., Tsai H.C., Chu C.T., Cheng H.W., Liu C.J., Lee C.H., Liu T.Y., Wang H.T. (2021). Cigarette Smoke Containing Acrolein Upregulates EGFR Signaling Contributing to Oral Tumorigenesis In Vitro and In Vivo. Cancers.

[194] Wong M., Martinez T., Hendricks N., Talbot P. (2025). Acetaldehyde and Methylglyoxal: Comparative Analysis of Toxic Electronic Cigarette Degradation Products in 3D and 2D Exposure Systems Using Human Bronchial Epithelial Models. Front. Toxicol..

[195] Ureña J.F., Ebersol L.A., Silakov A., Elias R.J., Lambert J.D. (2020). Impact of Atomizer Age and Flavor on In Vitro Toxicity of Aerosols from a Third-Generation Electronic Cigarette against Human Oral Cells. Chem. Res. Toxicol..

[196] Robertson N.E., Connolly J., Shevchenko N., Mascal M., Pinkerton K.E., Nicklisch S.C.T., Nguyen T.B. (2024). Chemical Composition of Aerosols from the Electronic Cigarette Vaping of Natural and Synthetic Cannabinoids. Chem. Res. Toxicol..

[197] Wong M., Martinez T., Tran M., Zuvia C., Gadkari A., Omaiye E.E., Luo W., McWhirter K.J., Sha J., Kassem A. (2023). A Synthetic Coolant (WS-23) in Disposable Electronic Cigarettes Impairs Cytoskeletal Function in EpiAirway Microtissues Exposed at the Air Liquid Interface. Sci. Rep..

[198] Ye D., Gajendra S., Lawyer G., Jadeja N., Pishey D., Pathagunti S., Lyons J., Veazie P., Watson G., McIntosh S. (2020). Inflammatory Biomarkers and Growth Factors in Saliva and Gingival Crevicular Fluid of Electronic Cigarette Users, Cigarette Smokers, and Dual Smokers: A Pilot Study. J. Periodontol..

[199] Hernandez A., Hedlich-Dwyer J., Hussain S., Levi H., Sonavane M., Suzuki T., Kamiya H., Gassman N.R. (2024). Acute Exposure to Dihydroxyacetone Promotes Genotoxicity and Chromosomal Instability in Lung, Cardiac, and Liver Cell Models. Toxicol. Sci..

[200] Lai S.W.T., Lopez Gonzalez E.J., Zoukari T., Ki P., Shuck S.C. (2022). Methylglyoxal and Its Adducts: Induction, Repair, and Association with Disease. Chem. Res. Toxicol..

[201] Kim M.D., Chung S., Baumlin N., Sun L., Silswal N., Dennis J.S., Yoshida M., Sabater J., Horrigan F.T., Salathe M. (2023). Electronic Cigarette Aerosols of Propylene Glycol Impair BK Channel Activity and Parameters of Mucociliary Function. Am. J. Physiol. Lung Cell. Mol. Physiol..

[202] Kwak S., Choi Y.S., Na H.G., Bae C.H., Song S.Y., Kim Y.D. (2021). Glyoxal and Methylglyoxal as Electronic Cigarette Vapor Ingredients-Induced Pro-Inflammatory Cytokine and Mucins Expression in Human Nasal Epithelial Cells. Am. J. Rhinol. Allergy.

[203] Azimi P., Keshavarz Z., Lahaie Luna M., Cedeno Laurent J.G., Vallarino J., Christiani D.C., Allen J.G. (2021). An Unrecognized Hazard in Electronic Cigarette Vapor: Preliminary Quantification of Methylglyoxal Formation from Propylene Glycol in Electronic Cigarettes. Int. J. Environ. Res. Public Health.

[204] Vijayraghavan S., Ruggiero A., Becker S., Mieczkowski P., Hanna G.S., Hamann M.T., Saini N. (2025). Methylglyoxal Mutagenizes Single-Stranded DNA via Rev1-Associated Slippage and Mispairing. Nucleic Acids Res..

[205] Retamal I.N., Hernández R., González-Rivas C., Cáceres M., Arancibia R., Romero A., Martínez C., Tobar N., Martínez J., Smith P.C. (2016). Methylglyoxal and Methylglyoxal-Modified Collagen as Inducers of Cellular Injury in Gingival Connective Tissue Cells. J. Periodontal Res..

[206] Park H.R., Vallarino J., O’Sullivan M., Wirth C., Panganiban R.A., Webb G., Shumyatcher M., Himes B.E., Park J.A., Christiani D.C. (2021). Electronic Cigarette Smoke Reduces Ribosomal Protein Gene Expression to Impair Protein Synthesis in Primary Human Airway Epithelial Cells. Sci. Rep..

[207] Jeong S.R., Lee K.W. (2021). Methylglyoxal-Derived Advanced Glycation End Product (AGE4)-Induced Apoptosis Leads to Mitochondrial Dysfunction and Endoplasmic Reticulum Stress through the RAGE/JNK Pathway in Kidney Cells. Int. J. Mol. Sci..

[208] Salamanca J.C., Meehan-Atrash J., Vreeke S., Escobedo J.O., Peyton D.H., Strongin R.M. (2018). Electronic Cigarettes Can Emit Formaldehyde at High Levels under Conditions That Have Been Reported to Be Non-Averse to Users. Sci. Rep..

[209] Yildiz D. (2004). Nicotine, Its Metabolism and an Overview of Its Biological Effects. Toxicon.

[210] Hukkanen J., Jacob P., Benowitz N.L. (2005). Metabolism and Disposition Kinetics of Nicotine. Pharmacol. Rev..

[211] Kanamori K., Ahmad S.M., Hamid A., Lutfy K. (2024). Chronic Exposure to Electronic Cigarettes Elevates CYP2A5 Activity, Protein Expression, and Cotinine-Induced Production of Reactive Oxygen Species in Mice. Drug Metab. Dispos..

[212] Venugopal P.D., Addo Ntim S., Goel R., Reilly S.M., Brenner W., Hanna S.K. (2024). Environmental Persistence, Bioaccumulation, and Hazards of Chemicals in Electronic Cigarette E-Liquids: Short-Listing Chemicals for Risk Assessments. Tob. Control.

[213] Margham J., McAdam K., Cunningham A., Porter A., Fiebelkorn S., Mariner D., Digard H., Proctor C. (2021). The Chemical Complexity of Electronic Cigarette Aerosols Compared with the Smoke from a Tobacco Burning Cigarette. Front. Chem..

[214] Chitteti R., Zuniga-Hertz J.P., Masso-Silva J.A., Shin J., Niesman I., Bojanowski C.M., Kumar A.J., Hepokoski M., Crotty Alexander L.E., Patel H.H. (2025). Electronic Cigarette-Induced Changes in Cell Stress and Mitochondrial Function. Free Radic. Biol. Med..

[215] Kasnak G., Könönen E., Syrjänen S., Gürsoy M., Zeidán-Chuliá F., Firatli E., Gürsoy U.K. (2019). NFE2L2/NRF2, OGG1, and Cytokine Responses of Human Gingival Keratinocytes against Oxidative Insults of Various Origin. Mol. Cell. Biochem..

[216] Al-Hamoudi N., Alsahhaf A., Al Deeb M., Alrabiah M., Vohra F., Abduljabbar T. (2020). Effect of Scaling and Root Planing on the Expression of Anti-Inflammatory Cytokines (IL-4, IL-9, IL-10, and IL-13) in the Gingival Crevicular Fluid of Electronic Cigarette Users and Non-Smokers with Moderate Chronic Periodontitis. J. Periodontal Implant Sci..

[217] Brunnemann K.D., Hoffmann D. (1991). Analytical Studies on Tobacco-Specific N-Nitrosamines in Tobacco and Tobacco Smoke. Crit. Rev. Toxicol..

[218] Li Y., Hecht S.S. (2022). Metabolism and DNA Adduct Formation of Tobacco-Specific *N*-Nitrosamines. Int. J. Mol. Sci..

[219] Shahab L., Goniewicz M.L., Blount B.C., Brown J., McNeill A., Alwis K.U., Feng J., Wang L., West R. (2017). Nicotine, Carcinogen, and Toxin Exposure in Long-Term Electronic Cigarette and Nicotine Replacement Therapy Users: A Cross-Sectional Study. Ann. Intern. Med..

[220] Du H., Leng J., Wang P., Li L., Wang Y. (2018). Impact of Tobacco-Specific Nitrosamine-Derived DNA Adducts on the Efficiency and Fidelity of DNA Replication in Human Cells. J. Biol. Chem..

[221] Lerner C.A., Sundar I.K., Yao H., Gerloff J., Ossip D.J., McIntosh S., Robinson R., Rahman I. (2015). Vapors Produced by Electronic Cigarettes and E-Juices with Flavorings Induce Toxicity, Oxidative Stress, and Inflammatory Response in Lung Epithelial Cells and in Mouse Lung. PLoS ONE.

[222] Hecht S.S. (1998). Biochemistry, Biology, and Carcinogenicity of Tobacco-Specific N-Nitrosamines. Chem. Res. Toxicol..

[223] Du X., Squier C.A., Kremer M.J., Wertz P.W. (2000). Penetration of *N*-Nitrosonornicotine (NNN) across Oral Mucosa in the Presence of Ethanol and Nicotine. J. Oral Pathol. Med..

[224] Xue J., Yang S., Seng S. (2014). Mechanisms of Cancer Induction by Tobacco-Specific NNK and NNN. Cancers.

[225] Kotandeniya D., Carmella S.G., Pillsbury M.E., Hecht S.S. (2015). Combined Analysis of *N*′-Nitrosonornicotine and 4-(Methylnitrosamino)-1-(3-Pyridyl)-1-Butanol in the Urine of Cigarette Smokers and Electronic Cigarette Users. J. Chromatogr. B Anal. Technol. Biomed. Life Sci..

[226] Bustamante G., Ma B., Yakovlev G., Yershova K., Le C., Jensen J., Hatsukami D.K., Stepanov I. (2018). Presence of the Carcinogen N′-Nitrosonornicotine in Saliva of Electronic Cigarette Users. Chem. Res. Toxicol..

[227] Chen K.M., Krebs N.M., Sun Y.W., Sun D., Liao J., Reinhart L., Mallery S.R., El-Bayoumy K. (2026). The Effects of Black Raspberry Lozenges on 4-Hydroxy-1-(3-Pyridyl)-1-Butanone-Releasing DNA Adducts in Buccal Cells and Urinary Levels of N′-Nitrosonornicotine and Its Glucuronide in Active Smokers. Carcinogenesis.

[228] Stanfill S.B., Hecht S.S., Joerger A.C., González P.J., Maia L.B., Rivas M.G., Moura J.J.G., Gupta A.K., Le Brun N.E., Crack J.C. (2023). From Cultivation to Cancer: Formation of *N*-Nitrosamines and Other Carcinogens in Smokeless Tobacco and Their Mutagenic Implications. Crit. Rev. Toxicol..

[229] Arredondo J., Chernyavsky A.I., Grando S.A. (2006). Nicotinic Receptors Mediate Tumorigenic Action of Tobacco-Derived Nitrosamines on Immortalized Oral Epithelial Cells. Cancer Biol. Ther..

[230] Stewart B.W., Marshall H., Bonevski B., Griffin H.J., Hopkins A.M., Itchins M., Mazza C.J., Modi N.D., Ryan M., Varlow M. (2025). The Carcinogenicity of Electronic Cigarettes: A Qualitative Risk Assessment. Carcinogenesis.

[231] Pettersen E.O., Nome O., Rønning O.W., Oftebro R. (1983). Effects of Benzaldehyde on Survival and Cell-Cycle Kinetics of Human Cells Cultivated In Vitro. Eur. J. Cancer Clin. Oncol..

[232] Zhang K., Wang H., Cheng S., Zhang C., Zhai X., Lin X., Chen H., Gao R., Dong W. (2021). A Benzaldehyde-Indole Fused Chromophore-Based Fluorescent Probe for Double-Response to Cyanide and Hypochlorite in Living Cells. Analyst.

[233] Muthumalage T., Lamb T., Friedman M.R., Rahman I. (2019). E-cigarette flavored pods induce inflammation, epithelial barrier dysfunction, and DNA damage in lung epithelial cells and monocytes. Sci. Rep..

[234] Lamb T., Rahman I. (2023). Pro-inflammatory effects of aerosols from e-cigarette-derived flavoring chemicals on murine macrophages. Toxicol. Rep..

[235] Ma T., Wang X., Li L., Sun B., Zhu Y., Xia T. (2021). Electronic Cigarette Aerosols Induce Oxidative Stress-Dependent Cell Death and NF-κB-Mediated Acute Lung Inflammation in Mice. Arch. Toxicol..

[236] Sachdeva J., Karunananthan A., Shi J., Dai W., Kleinman M.T., Herman D., Kloner R.A. (2023). Flavoring Agents in Electronic Cigarette Liquids: A Comprehensive Analysis of Multiple Health Risks. Cureus.

[237] Costantini E., Sinjari B., Falasca K., Reale M., Caputi S., Jagarlapodii S., Murmura G. (2021). Assessment of the Vanillin Anti-Inflammatory and Regenerative Potentials in Inflamed Primary Human Gingival Fibroblast. Mediat. Inflamm..

[238] Fechter-Leggett E.D., White S.K., Fedan K.B., Cox-Ganser J.M., Cummings K.J. (2018). Burden of Respiratory Abnormalities in Microwave Popcorn and Flavouring Manufacturing Workers. Occup. Environ. Med..

[239] Gerloff J., Sundar I.K., Freter R., Sekera E.R., Friedman A.E., Robinson R., Pagano T., Rahman I. (2017). Inflammatory Response and Barrier Dysfunction by Different Electronic Cigarette Flavoring Chemicals Identified by Gas Chromatography-Mass Spectrometry in E-Liquids and E-Vapors on Human Lung Epithelial Cells and Fibroblasts. Appl. Vitr. Toxicol..

[240] Foster M.W., Gwinn W.M., Kelly F.L., Brass D.M., Valente A.M., Moseley M.A., Thompson J.W., Morgan D.L., Palmer S.M. (2017). Proteomic Analysis of Primary Human Airway Epithelial Cells Exposed to the Respiratory Toxicant Diacetyl. J. Proteome Res..

[241] Muthumalage T., Prinz M., Ansah K.O., Gerloff J., Sundar I.K., Rahman I. (2018). Inflammatory and Oxidative Responses Induced by Exposure to Commonly Used e-Cigarette Flavoring Chemicals and Flavored e-Liquids without Nicotine. Front. Physiol..

[242] Fetterman J.L., Weisbrod R.M., Feng B., Bastin R., Tuttle S.T., Holbrook M., Baker G., Robertson R.M., Conklin D.J., Bhatnagar A. (2018). Flavorings in Tobacco Products Induce Endothelial Cell Dysfunction. Arterioscler. Thromb. Vasc. Biol..

[243] Whittaker P., Clarke J.J., San R.H., Begley T.H., Dunkel V.C. (2008). Evaluation of the butter flavoring chemical diacetyl and a fluorochemical paper additive for mutagenicity and toxicity using the mammalian cell gene mutation assay in L5178Y mouse lymphoma cells. Food Chem. Toxicol..

[244] Sun Y., Lin K., Effah F., Cartujano Barrera F., Li D., McIntosh S., McGraw M.D., Rahman I. (2025). Toxicity of Humectants Propylene Glycol and Vegetable Glycerin in Electronic Nicotine Delivery Systems. Toxicol. Lett..

[245] Tellez C.S., Juri D.E., Phillips L.M., Do K., Yingling C.M., Thomas C.L., Dye W.W., Wu G., Kishida S., Kiyono T. (2021). Cytotoxicity and Genotoxicity of E-Cigarette Generated Aerosols Containing Diverse Flavoring Products and Nicotine in Oral Epithelial Cell Lines. Toxicol. Sci..

[246] Ahmadi N., Zamanian Azodi M., Rostami Nejad M., Rezaei M., Robati R., Bandarian F., Razi F., Razzaghi Z., Arjmand B., Rezaei-Tavirani M. (2025). Evaluation of E-Cigarette Effects on Gene Expression Profile of Human Bronchial Epithelial Cells: A System Biology Approach. Tanaffos.

[247] Su L., Liu J., Yue Q., Zhang S., Zhao C., Sun X., Xu J., Jiang X., Li K., Li B. (2023). Evaluation of the Effects of E-Cigarette Aerosol Extracts and Tobacco Cigarette Smoke Extracts on Human Gingival Epithelial Cells. ACS Omega.

[248] Nobre da Silva J.L., Lima de Oliveira M., Matos da Penha G., Ferreira Moura M., Matias de Sousa A., Silveira Serra D., Gusmão Pinto Vieira Í., Feitosa Lima E.K., Ferreira Santos C., Falcão do Nascimento N.R. (2027). Caryocar coriaceum Fixed Oil Attenuates E-Cigarette Aerosol-Induced Pulmonary Injury and Oxidative Stress in Mice. J. Ethnopharmacol..

[249] Rinaldi S., Pieper E., Schulz T., Zimmermann R., Luch A., Laux P., Mallock-Ohnesorg N. (2023). Oral Nicotine Pouches with an Aftertaste? Part 2: In Vitro Toxicity in Human Gingival Fibroblasts. Arch. Toxicol..

[250] Ganapathy V., Chengizkhan G., Manyanga J., Chinnaiyan M., Sadhasivam B., Ramachandran I., Rubenstein D.A., Queimado L. (2026). Chronic E-Cigarette Aerosol Exposure Alters NRF2 and TGF-β1 Signaling and Enhances Stem-Like Features in Human Oral and Lung Epithelial Cells. Ecotoxicol. Environ. Saf..

[251] Joshi J., Pandit A., Shah F. (2023). Nicotine Mediated Epithelial Modulations: An In Vitro Evidence. J. Oral Biol. Craniofac. Res..

[252] Scott A., Lugg S.T., Aldridge K., Lewis K.E., Bowden A., Mahida R.Y., Grudzinska F.S., Dosanjh D., Parekh D., Foronjy R. (2018). Proinflammatory Effects of E-Cigarette Vapour Condensate on Human Alveolar Macrophages. Thorax.

[253] Chen I.L., Todd I., Tighe P.J., Fairclough L.C. (2020). Electronic Cigarette Vapour Moderately Stimulates Proinflammatory Signalling Pathways and Interleukin-6 Production by Human Monocyte-Derived Dendritic Cells. Arch. Toxicol..

[254] Wang Q., Sundar I.K., Li D., Lucas J.H., Muthumalage T., McDonough S.R., Rahman I. (2020). E-Cigarette-Induced Pulmonary Inflammation and Dysregulated Repair Are Mediated by nAChR α7 Receptor: Role of nAChR α7 in SARS-CoV-2 COVID-19 ACE2 Receptor Regulation. Respir. Res..

[255] Nguyen T., Nioi P., Pickett C.B. (2009). The Nrf2-Antioxidant Response Element Signaling Pathway and Its Activation by Oxidative Stress. J. Biol. Chem..

[256] Morgan M.J., Liu Z.G. (2011). Crosstalk of Reactive Oxygen Species and NF-κB Signaling. Cell Res..

[257] Hoffmann A., Cheng G., Baltimore D. (2025). NF-κB: Master Regulator of Cellular Responses in Health and Disease. Immun. Inflamm..

[258] Serdar C.C., Guney Z., Balci N., Altingoz S.M., Serdar M.A., Kurgan S. (2025). Salivary Trimethylamine N-Oxide: A Novel Non-Invasive Marker for Periodontal Inflammation. Clin. Oral Investig..

[259] Sedelnikova O.A., Redon C.E., Dickey J.S., Nakamura A.J., Georgakilas A.G., Bonner W.M. (2010). Role of Oxidatively Induced DNA Lesions in Human Pathogenesis. Mutat. Res..

[260] Lee H.W., Park S.H., Weng M.W., Wang H.T., Huang W.C., Lepor H., Wu X.R., Chen L.C., Tang M.S. (2018). E-Cigarette Smoke Damages DNA and Reduces Repair Activity in Mouse Lung, Heart, and Bladder as Well as in Human Lung and Bladder Cells. Proc. Natl. Acad. Sci. USA.

